# Assessment of ante mortem welfare indicators and the pathophysiology of captive-bolt trauma in equids at slaughter

**DOI:** 10.1017/awf.2024.70

**Published:** 2025-01-03

**Authors:** Katharine A Fletcher, Barbara Padalino, Martina Felici, Daniele Bigi, Georgina Limon-Vega, Andrew Grist, Troy J Gibson

**Affiliations:** 1 Animal Welfare Science and Ethics Group, Department of Pathobiology and Population Sciences, Royal Veterinary College, Hawkshead Lane, Hatfield AL9 7TA, UK; 2 Department of Agricultural and Food Sciences, University of Bologna, Viale Giuseppe Fanin 46, 40127 Bologna, Italy; 3 Faculty of Science and Engineering, Southern Cross University, East Lismore, NSW 2480, Australia; 4 Veterinary Epidemiology, Economics and Public Health Group, Department of Pathobiology and Population Sciences, Royal Veterinary College, Hawkshead Lane, Hatfield AL9 7TA, UK; 5 The Pirbright Institute, Woking GU24 0NF, UK; 6 Animal Welfare and Behaviour Group, School of Veterinary Sciences, University of Bristol, Langford BS40 5DU, UK

**Keywords:** Animal welfare, captive-bolt gun, *Equus caballus*, killing, post mortem, veterinary pathology

## Abstract

There is limited research into horse slaughter, particularly ante mortem welfare, and the effectiveness of captive-bolt gun (CBG) stunning, despite this being a widely used method worldwide. To address this evidence gap and explore associations between ante and post mortem factors, the welfare of 62 horses was assessed at a commercial Italian abattoir. Animal-based measures were used to identify stress-related behaviours and stunning effectiveness. A sub-sample (44%; 27/62) of heads were assessed for gross brain pathology. All animals in the study showed stress-related behaviours at all stages of the slaughter process. Additionally, 53% (33/62) of horses slipped in the stunning box, with poor floor surface condition and use of force associated with this. At least one sign of an ineffective stun was observed in 22% (14/62) of animals. Six animals were shot twice, with the application of a second shot significantly associated with a higher stress behaviour index score in the stunning box. Damage to critical brainstem structures was found in 85% (23/27) of heads that were assessed with gross pathology. An absence of damage to critical brainstem structures meant that animals were ten times more likely to show signs of ineffective stunning. These results highlight the risks to equine welfare throughout the slaughter process and suggest that mitigating ante mortem stress could improve stunning effectiveness, whilst CBG usage should be refined to ensure that critical brainstem structures are targeted.

## Introduction

Millions of equids are slaughtered each year across the world, with almost 30,000 horses *(Equus caballus)* in Italy alone (Zappaterra *et al.* 2022). Some horses arriving at Italian abattoirs have undergone reported journeys of up to 58 h (Zappaterra *et al.* 2022). There is the potential that transport can compromise welfare and heighten stress levels prior to arrival at the abattoir (Miranda-de la Lama *et al.* 2020; Nicolaisen *et al.* 2023). Previously, a high proportion of animals transported to slaughter in Europe have also been found to have injuries (Marlin *et al.* 2011) although this appears to have improved in recent years, potentially due to a mitigation of hazards and increased enforcement of transport standards (Messori *et al.* 2016; Zappaterra *et al.* 2022). Existing research into equine slaughter has focused primarily on transport to slaughter, reporting issues such as slips or falls when unloading and thermal discomfort (Grandin *et al.* 1999; Nivelle *et al.* 2020). There is a paucity of research into welfare once horses have arrived at the abattoir (Fletcher *et al.* 2022), due to the difficulties in obtaining these data at each stage of the slaughter process itself. Slips, falls and other injuries can be exacerbated when using coercive equipment such as whips and unnecessarily forceful handling (Dai *et al.* 2021). Such handling, coupled with other stressors within an abattoir environment, can increase negative emotions in the animals such as fear and stress (Nivelle *et al.* 2020; Dai *et al.* 2021).

Abattoir design and operation can both positively and negatively influence the experience of equids during the process, particularly in cases where the facility was originally designed for species other than equids, resulting in narrow corridors, inappropriate flooring, and small pens (Grandin *et al.* 1999). As a prey species, equids can be fearful of new environments and may display a flight or fight response in situations where they feel stressed, for example when isolated from conspecifics (Carroll *et al.* 2022; Fletcher *et al.* 2023). This can then present a risk to both the animal and abattoir staff. Therefore, facilities should be designed and constructed in a way as to minimise the risk of injuries, slips, falls or sudden noises (Council Regulation [EC] No 1099/2009 2009). However, there is a lack of scientific evidence that can inform policy and/or guidance on the welfare challenges equids may face prior to and during the slaughter process. Extrapolating findings from studies in other species might not be appropriate due to behavioural, physiological and physical differences between species.

One such welfare challenge is achieving a quick, humane, and effective kill. Free bullet rifle has been found to be an efficient method in horses (Gibson *et al.* 2015a). However, horses in Italy and many other countries tend to be stunned by penetrating captive-bolt gun (CBG) (Baier & Willson 2020), restrained in a stunning box, but usually without mechanical head restraint, prior to exsanguination. Of the limited research into this method, the results for horses have shown mixed effectiveness (Cáraves & Gallo 2007; Werner & Gallo 2008). Unlike free bullet rifle, CBG must be performed at point-blank range, to allow for maximum penetration of the bolt into the cerebral hemisphere to ensure that the animal is rendered insensible by the stun, with shot placement being the strongest determining factor for stunning effectiveness (Grist *et al.* 2019). However, this potentially causes accuracy difficulties for horses either unused to human handling or demonstrating reactive, stress behaviour and unable to be sufficiently restrained to enable correct point-blank gun placement. Guidance regarding positioning and effectiveness, and associated risk for ineffective stunning, has not necessarily been evidenced for equids. The Humane Slaughter Association (HSA) suggests a frontal shooting position of 20 mm above the intersection of lines drawn from the middle of each eye to the base of the opposite ear with the muzzle of the firearm angled towards the neck (HSA 2013). However, this is not based on published empirical evidence. Furthermore, an EC-funded report recognised that there was an absence of specific good practice guidance for horses (European Commission 2017).

This exploratory study aimed to assess equid welfare during commercial slaughter practices, and to identify associations between ante and post mortem factors. It was hypothesised that there would be an association between ante mortem welfare indicators and stunning effectiveness.

## Materials and methods

### Ethical approval

Ethical approval for this study was granted by the Royal Veterinary College, Clinical Research Ethical Review Board (reference URN 2022 2103-3). Consent was obtained from the owner of the abattoir, prior to data collection.

### Sample

A power analysis was conducted to determine the sample size to include in a five-day study for a target population estimated at 5,000 horses. Sample size focused on observing incomplete concussion and what factors are associated with this, with the expected proportion of incomplete concussion estimated at 10%, with 0.08% absolute precision and 95% confidence interval (CI). In the absence of published literature for equine welfare at both ante and post mortem, the expected proportion of welfare issues was obtained by averaging the proportions of severe injuries and animals with poor welfare and/or ineffective stunning, found by Gibson *et al.* 2015a,b (10%, 0% and 20.4%, respectively). The minimum sample size required was 60 horses.

### Data collection

The facility where data were collected was a commercial abattoir in Italy processing approximately 5,000 horses per annum. This abattoir principally slaughters horses and occasionally donkeys, mules, and cattle, although it was originally designed for only the latter species. Equid slaughter was conducted twice per week. Sixty-two horses were assessed during routine slaughter over a period of four non-consecutive days in July 2022.

Each animal’s date of arrival at the abattoir, country of origin (as reported in the passport, but not specific location within that country or other background information), sex, year of birth (as reported in the passport, but not specific date of birth), species and breed type (sports horse type, e.g. Thoroughbred/Standardbred; draught horse type, e.g. purpose-bred meat horse; native pony type, e.g. cob) were recorded for those being assessed. Animals were identified via their microchip number and by the order of slaughter so that the same animal could be traced through each stage (i.e. holding area, stunning box, bleeding area and post mortem).

The welfare assessment protocol used was developed following a systematic review of the literature (Fletcher *et al.* 2022) and combined the use of animal- and environmental-based measures. The protocol was first tested and refined in an abattoir in the UK. The protocol was then further field-tested during a preliminary pilot at the abattoir in Italy, with the accessibility and practicability of assessing each individual tested under these conditions and adapted accordingly.

**Figure 1.**
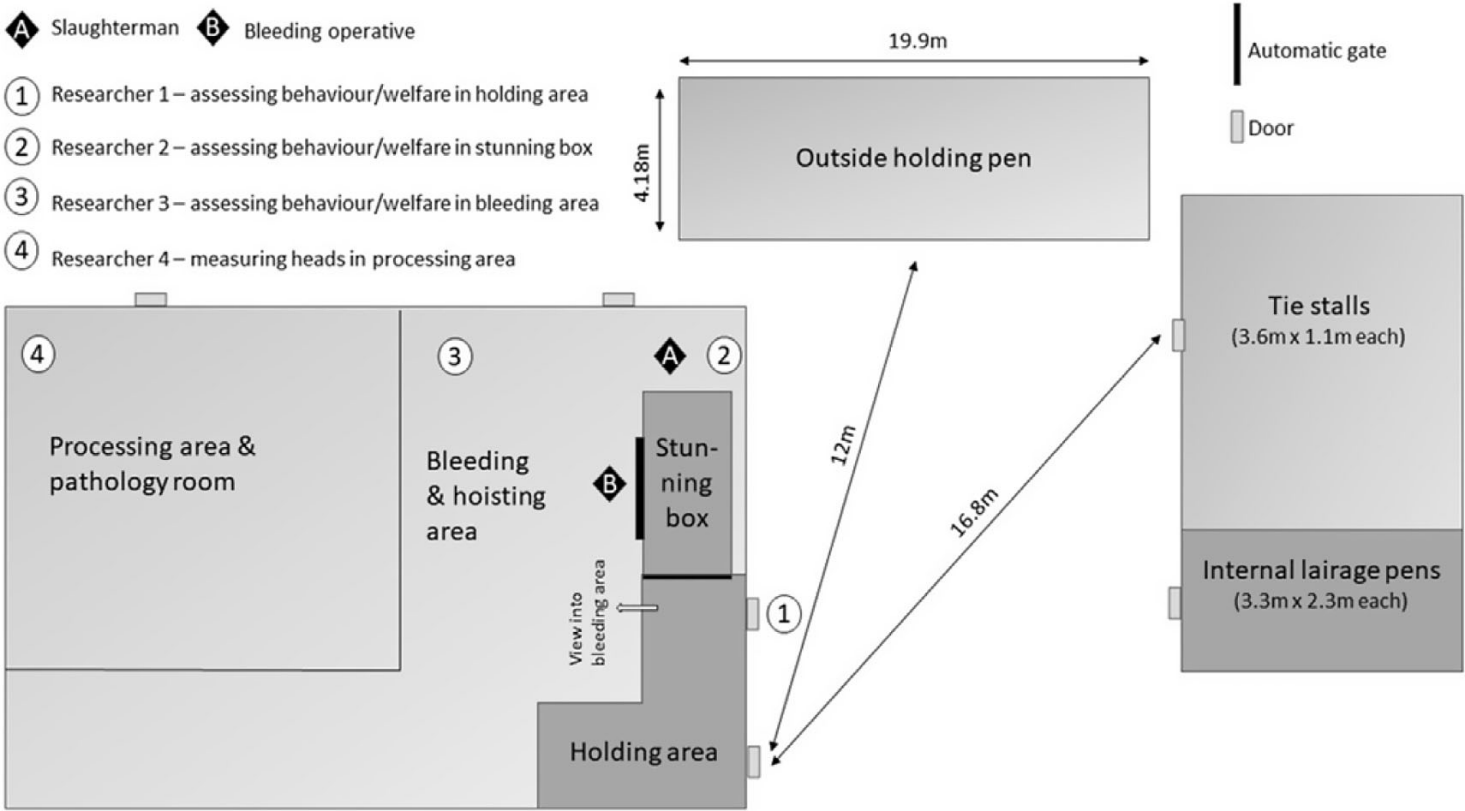
Diagram showing layout and positioning of research team members and slaughter operatives at a commercial abattoir in Italy where horses (n = 62) were studied at slaughter. NB diagram is not to scale.

The field team consisted of four assessors (KF, BP, MF, DB), all with behavioural and welfare assessment experience. Basic guidance was provided prior to the onset of data collection. Each team member was responsible for collecting data at one area throughout the study (Figure 1). Observations were recorded using Standardised recording sheets (see Supplementary material) with recording sheets either completed by hand or via a Dictaphone with a headset (Olympus VN-713PC, Olympus, Hachioji-shi, Tokyo, Japan) for subsequent transcription.

#### Ante mortem assessment

Lairage measurements including, lighting and visibility, were assessed by KF at the start of each day at the abattoir prior to data collection which was conducted at a similar time (approximately between 0600 and 0900h) each day. Lairage pen dimensions and stocking density were also recorded on the first day of data collection using a standardised recording sheet (Table 1).

**Table 1.** Lairage measurements and environmental indicators recorded on first day of data collection at a commercial abattoir in Italy where horses (n = 62) were studied prior to slaughter

Measurement	Scoring	Description
Stocking density	Adequate Inadequate	Ability to turn fully in the pen or have room for one horse’s length and width between one another (adapted from Raspa *et al*. (2020), as unable to accurately measure horse size/weight and this varied considerably)
Food Water Bedding Shelter	Present/Absent	
Cleanliness of pen/hazards	Good Moderate Poor	Minimal faeces and minimal/no hazards, e.g. rubbish Minor hazards and some faeces Multiple faeces and severe hazards
Vision/lighting	Good Moderate Poor	Well-lit with some natural light Some but either too bright/artificial or limited None
Air circulation/ventilation	Good Moderate Poor	Well ventilated Some but not reaching animal None/minimal

Distance from lairage to holding pens was measured using a laser distance meter (LV5800-50M, LOMVUM, Hangzhou, Zhejiang, China). On each day of data collection, immediately prior to the first horse being slaughtered, temperature and humidity were also measured using a thermometer and humidity meter placed inside the holding area (Kestrel 4000, Kestrel Instruments, Nielsen-Kellerman, Boothwyn PA, USA).

At the abattoir, on the morning of slaughter, animals were brought as a group from the lairage pens into a holding pen inside the main abattoir building where they would wait until being moved into the stunning box. On arrival in the holding pen, animals were observed from the moment they arrived at the front of the holding queue prior to entry to the stunning box, until the moment they entered the stunning box, and the door closed behind them. Whether animals required force/pressure to enter the stunning box, or whether they entered willingly, was recorded, along with whether they were restrained using a halter or were unrestrained/loose. There was a gap (no solid wall) just before the point at which the animal entered the stunning box, whereby they could see through into the bleeding and hoisting area (Figure 1). Assessors were positioned ≥ 1 m on one side of the animal, so as not to interfere with routine practice in the abattoir and to minimise observer effect. Only the right-hand side of the animal was able to be examined in the holding queue due to the position of the assessor. The total time observed was recorded using a digital stopwatch (Guang Cai Lun ZSD-809, Jeanoko, Longgang, Shenzhen, China) and entry into the stunning box was recorded as either willingly/minimal force, needing moderate pressure or needing significant pressure/force.

Animal-based measures – ocular discharge, nasal discharge, abnormal respiration, signs of disease/infection, skin/coat issues, lesions/wounds, scars/swellings, foot/limb abnormalities and signs of lameness – were recorded as present/absent. Body Condition Score was subjectively assessed through visual observation alone, using a five-point scale from 0 (emaciated) to 5 (obese) (Carroll & Huntington 1988).

Presence/absence (one-zero) recording of conspicuous behaviour was conducted for the total time the animal was observed at the front of the holding queue and for the total time that the animal was observed in the stunning box (Table 2). However, these times were variable and not of uniform length. Frequency and duration of behaviour were not recorded.

**Table 2.** Animal based measures (ABMs) recorded for each horse ante mortem in the holding pen and stunning box at a commercial abattoir in Italy where horses (n = 62) were studied prior to slaughter

Indicator/ABM	Description	Reference
Bite/bite threat	Grasps object, self or another animal in open mouth and bites	Regan *et al.* (2014)
Blinking	Closing movements of the eyelids	Fenner *et al.* (2016)
Calm and alert	Responding to surroundings e.g. ears moving and often forward	Burn *et al.* (2010)
Defaecation	Drops manure	Dai *et al.* (2020)
Dull/depressed	Less responsive to environment, often with glassy-eyed expression.	Torcivia & McDonnell (2021)
Ears back	Rotating ears to focus caudally or laying ears back against neck	Torcivia & McDonnell (2021)
Falls	A loss of balance, causing any part of the body (other than hooves) to touch the ground	Felici *et al.* (2022)
Head lowered	Moving or standing with the neck below horizontal	Torcivia & McDonnell (2021)
Head shaking	Rotational shaking of head	Torcivia & McDonnell (2021)
Hyper-responsive	Lower threshold and more animated reaction to stimuli	Torcivia & McDonnell (2021)
Increased respiration rate/rapid breathing	Rapid movements of the rib cage	Ayala *et al.* (2021)
Kick/kick threat	One or two legs are lifted and moved rapidly and forcefully	Dai *et al.* (2020)
Licking/chewing	The horse moves their tongue around their mouth and lips	Jaeger (2017)
Orienting towards a stimulus	Eyes/attention fixed intently on a stimulus (in case of stunning box: looking over the top of the front bar in the stunning box and staring intently in the direction of bleeding area)	Pearson *et al.* (2021) (adapted)
Pawing	Reaching a forelimb cranially while sweeping caudally	Torcivia & McDonnell (2021)
Pull back on halter	Backward pull against halter/lead rope pressure	-
Rear/attempt to rear	Rears with front legs	Dai *et al.* (2020)
Sclera visible	Wide-eyed, white of eye showing	Pearson *et al.* (2021)
Slips	A loss of balance, without any part of the body (other than hooves) touching the ground	Felici *et al.* (2022)
Sniffing ground	Sniffs the ground	Contreras-Aguilar *et al.* (2019)
Snort	Prolonged noisy exhalation	Regan *et al.* (2014)
Stomping	Flexing and then extending a limb, sharply striking hoof against ground	Torcivia & McDonnell (2021)
Sweating	Warm or damp, may include streams or droplets	Van Loon & Van Dierendonck (2019)
Trembling/shivering	Trembling, shivering or shaking	Pearson *et al*. (2021)
Turn head/avoidant	Moves or attempts to move away/turns head away	Burn *et al.* (2010)
Urination	Drops urine	Dai *et al.* (2020)
Vocalisation	Screaming/calling (emitting a long loud whinny vocalisation)	Torcivia & McDonnell (2021)
Weight shifting	Frequent shifting of the primary weight-bearing limb(s)	Torcivia & McDonnell (2021)
Yawning	An involuntary sequence consisting of mouth opening, deep inspiration, brief apnoea, and slow expiration	Padalino & Raidal (2020)

Human-animal interactions were assessed (Table 3) in the holding pen when the animal was at the front of the queue and then again in the stunning box prior to stunning.

**Table 3.** Human-Animal interactions, assessed both in the holding pen and stunning box for each horse at a commercial abattoir in Italy where horses (n = 62) were studied prior to slaughter

Indicator	Category	Description	Reference
Reaction to operator	Friendly/affiliative	Turns head towards/ears forward	Burn *et al.* (2010)
	Calm and alert	Responding to surroundings, e.g. ears moving and often forward, calm but not excessively aroused or hyper-responsive	
	Avoidant/nervous	Moves or attempts to move away/turns head away	
	Aggressive/agonistic	Attempts to bite, rear, kick or strike with foreleg; ears held back or flattened	
	Apathetic/depressed	Passive response to surroundings, e.g. head lowered	
Personnel vocalisations	Speaking	Speaks or whistles softly/quietly	Hultgren *et al.* (2014)
	Shouting	Speaks or shouts harshly/loudly	
	Rattling/slamming	Makes noise by clapping hands, slamming wall etc	
	Nothing of note/none		
Personnel attitude	Positive	Talking quietly, petting, touching	Waiblinger *et al.* (2002)
	Negative	Talking/shouting impatiently, forceful use of stick/hand	
	Neutral	Dominant talking, gentle touch with stick or hand	
Equipment used	Stick/whip		N/A
	None		
	Other	(e.g. hand, broom)	
Manner in which equipment used (if applicable)	Gentle	Soft and/or < 5×	Huertas *et al.* (2018)
	Intense	Stronger than before without damaging and/or > 5 – < 10×	
	Rough	Excessive force, damaging and/or > 10×	

#### Assessment at stunning and slaughter

Animals were restrained as per usual practice, either loose in the stunning box or using a halter with the rope held by the operator. Floor surface in the stunning box was subjectively measured throughout the day as poor (trip hazards and/or significant slip risks, e.g. faeces/wetness), moderate (minor trip hazards and/or some risk of slips/falls) or good (absence of trip hazards or wetness/faeces, unobstructed dry/level flooring).

Animals were shot with either a .22 or .25 penetrating Cash Special CBG (Accles & Shelvoke, Sutton Coldfield, UK), using the 2.5 gr (purple) or 3.0 gr (blue) cartridges, respectively. It was not possible for researchers to record which CBG/cartridge was used on each animal. All animals were shot by the same licenced slaughterman and the number of shot attempts was recorded.

One animal at time was usually loaded into the stunning box before being shot with a CBG. On just one occasion, two unhandled/unrestrained horses were loaded simultaneously in the box and the second horse was shot immediately after the first, prior to both then being released from the box. On that occasion, data were collected from both animals.

The stunned animal(s) were then ejected from the side of the box. Immediately after shooting, the animals were assessed for signs of effectiveness of stunning (adapted from Gibson *et al.* 2015a) with all variables recorded as binary: whether the indicator was present/absent (one-zero recording) (Table 4).

**Table 4.** Brainstem and behavioural signs of ineffective stunning assessed in each horse immediately post-stunning (adapted from Gibson *et al.* 2015a) at a commercial abattoir in Italy where horses (n = 62) were studied at slaughter

Animal-based measures	Description
Immediate collapse	Animal collapses immediately after the shot
Righting reflex	Makes co-ordinated effort to stand or lift head
Vocalisation	Vocalises independently from exhalation
Rhythmic breathing	Ribcage continuously moves in and out rhythmically
Gasping	Spasmodic sharp intake of breath with the mouth open
Leg kicking (convulsions)	Uncontrolled involuntary kicking movements
Eyeball rotation	Eyes rotated, not central, sclera visible
Spontaneous blinking	Opens/closes eyelid without stimulation
Nystagmus	Rapid involuntary movements of the eye
Palpebral reflex	Involuntary blink reflex when the medial canthus is stimulated
Corneal reflex	Involuntary blink reflex when cornea is stimulated
Muscle spasms	Absence of tonus in body/excessive muscle activity
Response to ear pinch	Pinching ear tip is followed by pain reaction/withdrawal
Response to nostril pinch	Pinching nasal septum is followed by pain reaction/withdrawal
Nostrils flaring	Movements/trembling of the nostrils
Response to cut/knife	Body or head movements during sticking procedure

A second slaughterman shackled one hindleg and hoisted the animal onto a bleeding rail. Each animal was bled by ventral neck incision which was generally conducted once the animal was shackled and hoisted. Additionally, some horses received a facial cut (of the transverse facial artery) prior to ventral neck incision, which was generally conducted immediately following ejection from the stunning box, with the operator cutting the carotid arteries caudal to the guttural pouch. The time from stun to both cuts was recorded (if applicable). Signs of ineffective stunning were not assessed once bleeding had begun.

#### Post mortem assessment

A sub-sample of 27 heads (44%; 27/62) were assessed (selected at random, based on the number of heads that could be processed across the four days of data collection), post mortem. This was conducted *in situ* at the abattoir once the heads had been removed and skinned as part of routine abattoir processing. The length of the head from the top of the poll to the tip of the nasal plane was measured, along with the width of the head from the widest point of each eye and the distance from shot position to the tip of the nasal plane. The shot entry position was first determined by placing transparent acetate over the head, with the HSA’s frontal shooting position (the middle of the forehead, 20 mm above the intersection of lines drawn from the middle of each eye to the base of the opposite ear with the muzzle of the firearm angled towards the neck (HSA 2013) marked on the acetate as ‘0’. The actual point of entry, sagittal and lateral, was also marked, with the difference between them (deviation from the HSA’s position) measured. The angle of the bolt through the brain was measured using a protractor (No 44, Moore & Wright, Sheffield, UK) with a metal probe inserted into the shot hole to measure the angle of entry. After measuring, each head was sawn longitudinally through or close to the bolt entry site. Heads were then examined for skull thickness, using a digital vernier calliper (Louisware model-2, B01MAY5ECH, Shenzhen, Guangdong, China) and for visual evaluation of entrance wounds, bolt trajectory, fractures, haemorrhage and damage to brain regions. Shots were assessed as having missed the brain when the bolt or associated bone fragments failed to enter the cranial vault.

The brains were examined *in situ* and then removed and sliced into sections approximately 7–10 mm (subjectively estimated visually). The sawing process was estimated to take away approximately 1–2 mm of brain tissue with some tissue dislodged. All brains were assessed immediately *in situ* at the abattoir, with photographs taken with a digital camera (Olympus IM015 TG-6, Olympus, Hachioji-shi, Tokyo, Japan) of the brain at each stage of analysis, to allow for retrospective confirmation of details, with distance standardised within approximately 150 mm of the brain itself.

The brains were then examined for gross macroscopic damage, displacement of tissues, haemorrhage (in the third ventricle, lateral ventricles, cerebral aqueduct, fourth ventricle, subarachnoid) and petechial haemorrhage, cavitation of the skull and position of bone and skin fragments. Haemorrhage over the entire brain surface was assessed subjectively as a percentage of the overall brain surface area. Data from the left and right hemispheres were pooled to aid analysis. Severity of tissue damage to specific brain regions (occipital, temporal, parietal and frontal lobes, thalamus, midbrain, pons, medulla, cerebellum, and spinal cord) was assessed subjectively as none (0%), mild (1–20%), moderate (21–49%) and severe (≥ 50%) (Gibson *et al.* 2015a,b; Costa *et al.* 2020).

#### Data handling and statistical analysis

Results were entered onto a Microsoft® Excel® (Version 2008) spreadsheet by KF. Data were coded for analysis, with age, floor condition, breed, human-animal interactions (personnel vocalisations, attitude and animal’s reaction to operator), deviation from the HSA’s shooting position and behavioural/brainstem signs of consciousness post-stun (ineffective stun) re-categorised (Table 5). Behavioural scores were presented as an ‘overall score’, by summation of the number of stress behaviours present (hyper-responsive, orienting towards a stimulus, ears back, head shaking, pull back on halter, turn head/avoidant, head lowered, sniffing ground, sclera visible, blinking, yawning, licking/chewing, vocalisation, snort, bite/bite threat, kick/kick threat, stomping, pawing, weight-shifting, trembling/shivering, urination, defaecation, rear/attempt to rear, increased respiration/rapid breathing). Positive behaviours (i.e. calm and alert) were not included in calculation of the overall score. Each behaviour was weighted equally. Repeated measures were not conducted, with overall behavioural scores assessed for all animals at each stage rather than individually (holding pen and stunning box). The operators for each stage (holding pen and stunning box) were also different, and so analysis was not conducted to assess associations between stages for human-animal interactions.

**Table 5.** Recategorisation of data for further statistical analysis at a commercial abattoir in Italy where horses (n = 62) were studied prior to slaughter

Variable	Initial categorisation	Re-categorisation
Age	Numerical	< 2.5 years old (born since 2020)
		> 2.5 years old to ≤ 6 years old (born between 2016 and 2019)
		> 6 years old to ≤ 12 years old (born between and 2010 and 2015)
		> 12 years old to ≤ 21 years old (born between 2000 and 2009)
		> 21 years old (born 1999 or before)
Floor condition	Good Moderate Poor	Good Poor (Moderate/Poor)
Deviation from HSA shooting position *(20 mm above the intersection of a line drawn from the middle of each eye to the base of the opposite ear)*	Numerical	≤ 10 mm = no deviation > 10 mm = deviation
Effective/ineffective stunning	Presence/absence of: Immediate collapse Rhythmic breathing Spontaneous blinking Corneal reflex Palpebral reflex Nystagmus Eyeball rotation Gasping Leg kicking Muscle spasms Righting reflex Response to ear and/or nostril pinch Vocalisation Nostril flaring Response to cut/knife	Animals were classified as ineffectively stunned if they failed to collapse or rhythmic breathing was present or if > 2 of the following parameters were present: Corneal reflex Palpebral reflex Eyeball rotation Nystagmus
Personnel vocalisations	Speaking Shouting Rattling/slamming Nothing of note/none	Silent/calm (speaking and/or nothing of note/none) Shout/slam (shouting and/or rattling/slamming)
Personnel attitude	Neutral Negative Positive	Neutral or positive Negative
Reaction to operator	Friendly/affiliative Avoidant/nervous Calm and alert Aggressive/agonistic Apathetic/depressed	Positive (friendly/affiliative and/or calm and alert) Negative (avoidant/nervous and/or aggressive/agonistic and/or apathetic/depressed)
Entry to stunning box	Minimal/mild pressure Moderate force Significant force	No/minimal force Force (Moderate and significant)
Equipment used	Stick/whip Other (hand/body shove) None	Equipment used (including body/hand) No equipment used
Restraint	Unrestrained/loose Mild pressure, no pulling Moderate pressure Significant pressure	Unrestrained Restrained (halter with mild, moderate or significant pressure)

Animals were classified as ineffectively stunned after CBG stunning if they failed to collapse and/or rhythmic breathing was present and/or if at least two of the following parameters were present: positive corneal reflex, positive palpebral reflex, eyeball rotation and nystagmus (Table 5) (Gibson *et al.* 2015a,b).

The distribution of continuous data was evaluated through frequency histograms. Descriptive statistics were performed, and non-normally distributed data were expressed through median and interquartile range (IQR) and normally distributed data were expressed through mean (± SD).

Related/associated samples, e.g. behaviours most commonly expressed at each stage by individual horses, were compared using McNemar Chi-squared tests. Chi-squared (or Fishers Exact as appropriate) tests were performed for independent samples to determine if there was an association between each behaviour variable and explanatory variables: (i) floor condition; (ii) human-animal interactions; (iii) number of shots; and (iv) effective/ineffective stunning. Differences between behavioural scores and stunning effectiveness were explored through Mann-Whitney *U* tests. Where numbers allowed (and where required, categories were combined to enable this, as per Table 5), univariate/logistic regression was then conducted with the above five categories as explanatory/predictor variables and each behaviour assessed following stunning (Table 4) as an outcome. Significant outcomes between predictor variables (e.g. force used) were checked for collinearity and when present only one (the one with lower *P*-value) was kept for further multivariable analysis. Odds ratios (OR) and 95% confidence interval (CI) were calculated as measures of strength of association. SPSS® (IBM SPSS® Statistics 28.0.0.0, 2022) was used for all statistical analysis and *P* ≤ 0.05 was the indicator of significance.

## Results

### Ante mortem assessment

#### Descriptive statistics

The mean (± SD) ambient temperature over the course of data collection was 28.6 (± 1.8)^o^C (range: 27.0–30.5^o^C), with mean humidity at 53 (± 0.04)%. The mean size of the lairage pen was 7.2 m × 2.6 m (length × width) (interquartile range [IQR]: 2.9–19.9 m × 1.1–4.2 m). Of the five different lairage areas assessed at the start of data collection on the first day, stocking density was scored as adequate on all days throughout the study period. Air circulation/ventilation and vision/lighting was scored as good in two areas, and moderate in three areas. Cleanliness of pen/floor surface was scored as good in one area and as moderate in four areas. Food, water, bedding and shelter were present in all but one lairage pen, which was the outside pen in which animals waited directly prior to being brought in for slaughter.

Most animals were born in 2016 (range 1992–2022) making the median age 6 years (IQR: 1–4 years), with 42% (25/60) of animals less than 2.5 years old (Table 6). Fifty-seven percent of animals (34/60) had been transported from France, with 41% (14/34) of these being sports horse types compared to 31% overall (19/62). Animals spent between one and eleven days at the abattoir prior to slaughter, with a mean of 4.1 (± 2.8) days.

**Table 6.** Demographics of horses included in sample at a commercial abattoir in Italy where horses (n = 62) were studied prior to slaughter

Variable	Proportion % (n)
*Age*	
< 2.5 years old (born since 2020)	42% (25/60)
> 2.5 years old to ≤ 6 years old (born between 2016 and 2019)	10% (6/60)
> 6 years old to ≤ 12 years old (born between and 2010 and 2015)	12% (7/60)
> 12 years old to ≤ 21 years old (born between 2000 and 2009)	30% (18/60)
> 21 years old (born 1999 or before)	7% (4/60)
Missing data	3% (2/62)
*Breed type*	
Draught horse type	34% (21/62)
Native pony type	35% (22/62)
Sports horse type	31% (19/62)
*Sex*	
Male	51% (30/59)
Female	49% (29/59)
Missing data	5% (3/62)
*Country of origin*	
France	57% (34/60)
Poland	17% (10/60)
Italy	12% (7/60)
Hungary	5% (3/60)
Czech Republic	5% (3/60)
Slovenia	3% (2/60)
Romania	1% (1/60)
Missing data	3% (2/62)

*Total percentages: Note that total percentages do not always sum to 100% for every characteristic due to rounding.*

Animals were observed in the holding pen for a mean time of 137.5 (± 98.6) s (min–max: 19–668 s). The median Body Condition Score was 3 (IQR: 2–3). Fifty percent (31/62) of animals showed ocular discharge, 48% (30/62) showed skin/coat issues, 45% (28/62) had lesions or wounds and 35% (22/62) had nasal discharge. Ten percent (6/62) of animals had all four of these and 23% (14/62) had both ocular and nasal discharge.

There was a significant association between overall behavioural score for the holding pen and stunning box (*P* = 0.001). The median behaviour score was 7 (IQR: 5–9; min–max: 1–14) and 8 (IQR: 6–10; min–max: 2–15) for the holding pen and stunning box, respectively. The behaviours more often shown by horses were orienting towards a stimulus, visible sclera, hyper-responsiveness, pulling back on halter, and licking/chewing (Table 7). Additionally, 23% (14/62) horses were seen to slip in the holding pen.

**Table 7.** Behaviours most frequently observed in holding pen and stunning box at a commercial abattoir in Italy where horses (n = 62) were studied prior to slaughter. The *P*-values from a McNemar Chi-squared test indicate if there are significant differences (*P* ≤ 0.05) between the holding pen and stunning box for each behaviour, for each individual animal. Significant values shown in bold

Behaviour	Holding pen% (n)	Stunning box% (n)	*P* value
Orienting towards stimulus	76% (47/62)	72% (44/62)	0.68
Sclera visible	73% (45/62)	81% (50/62)	0.38
Hyper-responsive	65% (40/62)	73% (45/62)	0.36
Pull back on halter	61% (38/62)	16% (10/62)	**< 0.001**
Licking/chewing	60% (37/62)	24% (15/62)	**< 0.001**
Head lowered	50% (31/62)	10% (6/62)	**< 0.001**
Increased respiration rate/rapid breathing	45% (28/62)	63% (39/62)	0.09
Sniffing ground	42% (26/62)	65% (40/62)	**0.007**
Turn head/avoidant	42% (26/62)	77% (48/62)	**< 0.001**
Vocalisation	40% (25/62)	31% (19/62)	0.24
Trembling/shivering	32% (20/62)	44% (27/62)	0.22
Pawing	21% (13/62)	11% (7/62)	0.18
Ears back	19% (12/62)	84% (52/62)	**< 0.001**
Weight shifting	16% (10/62)	31% (19/62)	0.09
Snort	16% (10/62)	10% (6/62)	0.42
Blinking	5% (3/62)	58% (36/62)	**< 0.001**

Animals were observed in the stunning box for a mean time of 81.6 (± 80.4) s, (min–max: 23–490 s). In the holding pen, significantly more horses pulled back on the halter, showed licking/chewing and low head carriage, compared to in the stunning box (*P* < 0.001). Whilst in the stunning box significantly more horses sniffed the ground (*P* = 0.007), turned their head away (avoidance) (*P* < 0.001), had ears backwards (*P* < 0.001), and rapid blinking (*P* < 0.001), compared to the holding pen. The behaviours most often shown by horses in both the holding pen and stunning box were orienting towards a stimulus, visible sclera, and hyper-responsiveness (Table 7). Additionally, 53% (33/62) horses were seen to slip in the stunning box.

Ninety percent of animals (56/62) were restrained (i.e. haltered). Force was used for 45% (28/62) of animals to enable entry to the stunning box, with a stick used in 84% (52/62) of cases. Operators shouted in the holding pen more often than the operator in the stunning pen, who was anecdotally perceived as being fairly silent with a neutral attitude (Table 8).

**Table 8.** Human-animal interactions observed in both the holding pen and stunning box at a commercial abattoir in Italy where horses (n = 62) were studied prior to slaughter

Human-animal interactions	Holding pen% (n)	Stunning box% (n)
*Personnel vocalisations*		
Shouting	74% (46/62)	5% (3/62)
Speak softly/calmly	24% (15/62)	24% (15/62)
Slamming/rattling	2% (1/62)	0% (0)
Nothing of note/none	0% (0)	71% (44/62)
*Personnel attitude*		
Neutral	89% (55/62)	82% (51/62)
Negative	6% (4/62)	16% (10/62)
Positive	5% (3/62)	2% (1/62)
*Reaction to operator*		
Friendly/affiliative	61% (38/62)	2% (1/62)
Avoidant/nervous	26% (16/62)	66% (41/62)
Calm and alert	11% (7/62)	29% (18/62)
Aggressive/agonistic	2% (1/62)	0% (0)
Apathetic/depressed	0% (0)	3% (2/62)
*Entry to stunning box*		
Minimal/mild pressure	56% (35/62)	-
Moderate force	39% (24/62)	-
Significant force	6% (4/62)	-
*Equipment used*		
Stick/whip	84% (52/62)	-
Other (hand/body shove)	8% (5/62)	-
None	11% (7/62)	-
*Manner in which equipment used*		
Gentle	71% (44/62)	-
Intense	18% (11/62)	-
Rough	0% (0/62)	-
Not applicable	11% (7/62)	

Horses were more likely to show a negative (avoidant/nervous and/or aggressive/agonistic and/or apathetic/depressed) response to the operator in the holding pen if they were unrestrained (*P* = 0.05, OR: 6.0, 95% CI: 1.0–36.4) compared to being restrained in a halter. Horses who had a higher overall behavioural score in the holding pen were more likely to show a negative response to the operator in the stunning box (*P* = 0.04, OR: 1.3, 95% CI: 1.0–1.6). No other human-animal interactions had a significant association with behaviour/welfare indicators.

Univariate analysis found that slips were significantly associated with floor condition (*P* = 0.014, OR: 3.9, 95% CI: 1.3–11.3), with horses more likely to slip if the floor surface was categorised as poor, rather than good. No horses were seen to slip when the floor condition was categorised as good, with 53% (33/62) of horses slipping on a poor floor surface.

Significantly more horses were likely to slip in the stunning box if force to assist with entry was used (*P* = 0.04, OR: 3.0, 95% CI: 1.1–8.6) and if personnel shouted (*P* = 0.047, OR: 3.4, 95% CI: 1.0–11.6). There was strong collinearity between personal shouting and force used, therefore only personnel shouting was considered in the multivariable analysis. Multivariable analysis found that significantly more horses slipped in the stunning box if the floor condition was poor (*P* = 0.008, OR: 6.0, 95% CI: 1.6–22.9), and if personnel shouted in the holding pen, rather than speaking calmly or not vocalising to the animals at all (*P* = 0.045, OR: 4.9, 95% CI: 1.0–23.1).

### Assessment at stunning/slaughter

Leg kicking/convulsions were seen in 91% (57/62) of horses after stunning and muscle spasms were seen by 23% (16/62). Twenty-two per cent of animals (14/62), showed signs of ineffective stunning (Table 9, and see Table S1 [Supplementary material]). All 14 animals were rhythmically breathing, although assessment of this was complicated by post-stun kicking. Six animals were shot twice (10%; 6/62) but could not be examined after the first shot, the time between shots was only logged for three animals (31, 40, and 74 s), with the remaining three animals shot again within a few seconds. One animal which was shot twice did not display signs of an ineffective stun in accordance with the study criteria but did show a response to nostril pinching. A seventh animal was shot twice but due to the gun misfiring the first shot did not penetrate the skin hence this animal was not included in analysis of those shot twice. Of the nine animals showing signs of ineffective stunning but not receiving a second shot, all displayed rhythmic respiration, three showed nystagmus, one showed eyeball rotation but not nystagmus, and this same animal also attempted to right itself. None of these nine showed corneal or palpebral reflex or failed to immediately collapse (Table 9).

**Table 9.** Signs of ineffective stunning observed in horses after first shot at a commercial abattoir in Italy where horses (n = 62) were studied at slaughter

Behaviour/signs	% (n)(Total = 62)	Missing data	Of which shot twice(10%, 6/62)
Rhythmic breathing	22% (14/62)	0	5
Possible righting reflex^†^	19% (12/62)	0	3
Nystagmus*^‡^	18% (10/56)	6	3
Nostrils flaring	15% (9/62)	26	3
Eyeball rotation*^‡^	7% (4/56)	6	3
Response to cut/knife	8% (5/62)	19	1
Gasping	6% (4/62)	0	0
Spontaneous blinking*^‡^	5% (3/56)	6	1
Corneal reflex*^‡^	3% (2/56)	6	2
Palpebral reflex*^‡^	3% (2/56)	6	2
Lack of immediate collapse	3% (2/62)	0	2
Response to nostril pinch*	2% (1/57)	6	1
Vocalisation	2% (1/62)	0	1
Response to ear pinch*	0% (0)	6	0

* n = 6 animals could not be checked for these responses due to assessor safety

† n = 5 also showed excessive leg kicking which complicated assessment of righting reflex.

‡ Only one eye was assessed

Overall behavioural score in the holding pen was not significantly associated with whether the animal was then perceived to be effectively stunned or not (*P* = 0.18). However, univariate regression analysis found that there was a trend towards a higher behavioural score in the stunning box tending to result in an ineffective stun (*P* = 0.06; OR: 1.2, 95% CI: 1.0–1.5). Combined overall behavioural score for the holding pen and stunning box was not significantly associated with whether the animal received a single shot, or a repeated shot (*P* = 0.19). However, a higher behavioural score in the stunning box increased the odds of a second shot being needed (*P* = 0.005; OR: 2.2, 95% CI: 1.3–2.8).

A total of 25 animals received a facial arterial cut, of which 32% (8/25) were sports horse types, 56% (14/25) were native pony types and 12% (3/25) were draught horse type, with the median time from stunning to facial cutting 18 s (IQR: 24–14; min–max: 10–52 s). The median time from stunning to the ventral neck cut was 69.0 s (IQR: 61–80) for all animals (min–max: 34–130 s). For those animals that received a facial cut (n = 25), the median time from stunning to the ventral neck cut was 70.0 s (IQR: 81.5–64; min–max: 48–130) s, compared to those that did not receive a facial cut, where it was 68.5 s (IQR: 78.5–60; min–max: 34–105) s. However, this was not significantly different (*P* = 0.89).

### Post mortem assessment

**Figure 2.**
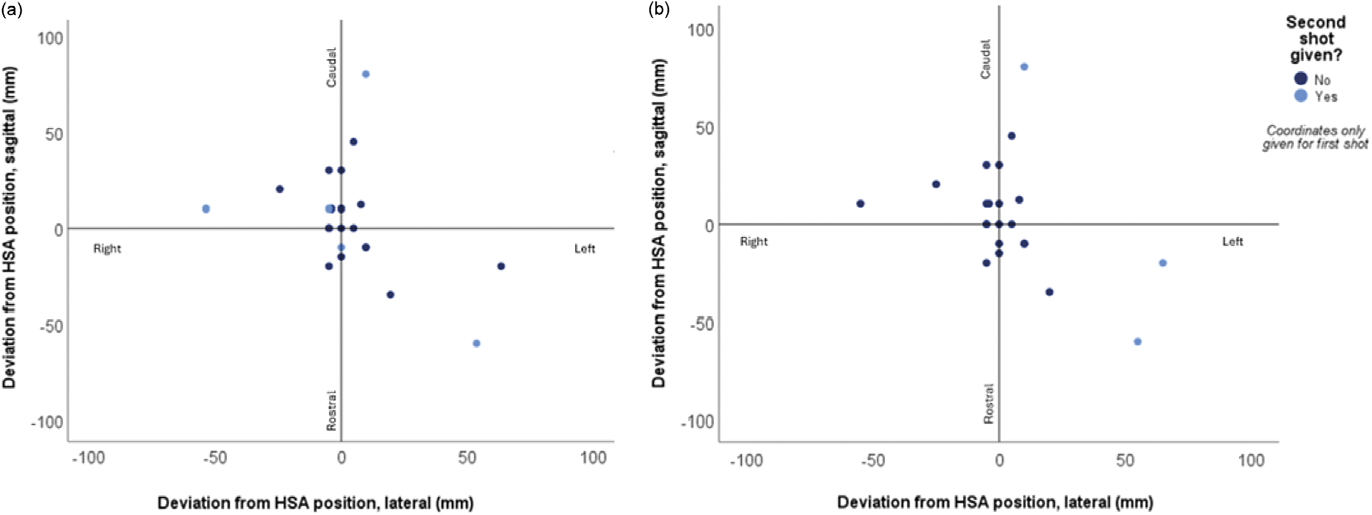
Scatterplot showing deviation from the suggested (HSA [Humane Slaughter Association] 2013) position for captive-bolt shooting of horses (– is left from operator’s perspective and rostral of midline), showing (a) where animals showed signs of effective or ineffective stunning and (b) if a second shot was given (n = 23). These originated from a commercial abattoir in Italy where horses (n = 62) were studied at slaughter.

Post mortem assessment was performed on a subset of 43% (27/62) animals. Due to missing data, deviation from the HSA shooting point (20 mm above the intersection of a line drawn from the middle of each eye to the base of the opposite ear) was assessed in 85% (23/27) of these (Figure 2). Forty-three percent of these animals (10/23) were assessed as having no deviation (≤ 10 mm sagittal and/or lateral) from the HSA shooting position. Two of these animals still showed signs of ineffective stunning (rhythmic respiration), they were challenging to assess due to leg kicking and one could not have their eye reflexes assessed. Both were assessed as having damage to critical brain structures but neither had damage to the medulla. Head and shot measurements are detailed in Table 10. Fifty-seven percent were shot at more than 10 mm deviation and 39% at more than 20 mm deviation, with the maximum deviation 80 mm caudally and most shots to the right of the HSA position. Ineffective stunning was determined in only 31% of animals with more than 20 mm deviation.

**Table 10.** Measurements taken for horse head size, deviation from the Humane Slaughter Association (HSA 2013)’s shooting position, and angle of shot, for the first shot only (n = 23), with ‘–’ indicating left of midline and ‘+’ indicating right of midline at a commercial abattoir in Italy where horses were studied at slaughter

Variable	Mean	SD	Min	Max
Deviation from HSA position, sagittal (mm)	3.4	27.2	–60	+80
Deviation from HSA position, lateral (mm)	3.6	22.9	–55	+65
Length of head (mm)	584.8	35.7	510	660
Width of head (widest point of eyes) (mm)	197.4	22.9	170	290
Distance from shot entry to tip of nasal bone (mm)	456.6	36.0	370	560
Distance between inner corner of eyes (mm)	249.6	14.9	220	290
Angle of shot transverse (°)	83.3	6.7	65	96
Angle of shot sagittal (°)	84.9	10.4	60	105

Skull thickness was assessed for 20 animals (seven could not have skull thickness assessed due to saw damage) and ranged between 5 and 16 mm, with a mean (± SD) of 9.0 (± 3.0) mm. There was no significant difference in mean skull thickness for sports horse types (9.0 ± [2.8] mm), native types (7.2 [± 1.1] mm) and draught types (12.1 [± 3.1] mm) (*P* = 0.09). The parietal lobe was the most common point of entry, with 63% (17/27) of shots assessed entering through this lobe, 19% (5/27) entering at the frontal lobe, 11% (3/27) through the occipital lobe, and 4% (1/27) at the temporal lobe.

**Figure 3.**
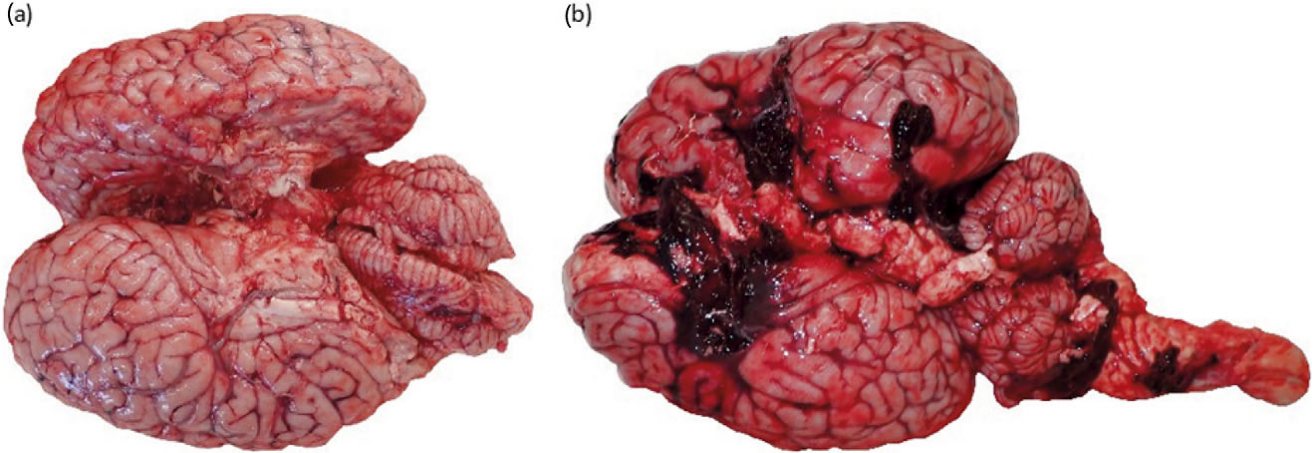
Showing (a) the brain of a horse which showed signs of ineffective stunning (rhythmic respiration) and no macroscopic brain injury (did not receive a second shot). The shot was 10 mm rostral and 55 mm left lateral of the suggested shot position (HSA [Humane Slaughter Association] 2013) missing the brain and (b) the brain of a horse irrecoverably stunned. This horse displayed no signs of consciousness, it was shot in the parietal lobe with severe damage to the parietal lobe, mild damage to the frontal lobe, temporal lobe and midbrain, and moderate damage to the thalamus, with the shot having been 10 mm rostral and 4 mm right of the suggested shot position (HSA 2013). These were part of a study at a commercial abattoir in Italy where horses (n = 62) were studied at slaughter.

Six animals who were examined for gross pathology showed signs of ineffective stunning. Two of these animals were also found to have live maggots in the ethmoidal concha. These two animals were also considered to be semi-feral draught horse types, unaccustomed to human handling and more likely to move in the stunning box. In both animals, the first shot was off-centre (10 to 80 mm rostral, –20–65 mm and 80 mm caudal, respectively, from the HSA suggested position). In the first case, the shot had missed the brain (Figure 3) and in the second case, the horse was shot on the left-hand side of the cranial vault, and it had not penetrated the cranial vault but had caused bone fragments to penetrate at a right angle to the bolt into the temporal lobe. There was no damage to the thalamus, midbrain, pons or medulla in either case. Of the remaining four animals which showed signs of ineffective stunning, one shot had not penetrated the brain and had not caused any damage to cerebral lobes, thalamus, midbrain, or brainstem structures (Table 11). In the other three animals where the bolt entered the cranial vault, there was no damage to midbrain or brainstem structures, apart from one horse that had moderate damage to the midbrain but not damage to the brainstem. Two of these animals also had moderate to severe damage to the thalamus (Figure 3).

**Table 11.** Level of macroscopic damage to specific brain structures and signs of consciousness observed post-stun for each horse (total n = 27) at a commercial abattoir in Italy where horses were studied at slaughter

Level of macroscopic brain damage to each region (%; n)	Post-stunning behaviour shown (%, n)						
	Legkicking(96%; 26/27)	Nystagmus(11%; 3/27)	Eyeballrotation(11%; 3/27)	Rhythmic breathing(22%; 6/27)	Spontaneous blinking(3%; 1/27)	Failure to collapse(7%; 2/27)	Rightingreflex(22%; 6/27)
Frontal lobe: (55%, 15/27)							
- None	(44%; 11/25)	(0%; 0/3)	(33%; 1/3)	(66%; 4/6)	(0%; 0/1)	(50%; 1/2)	(33%; 2/6)
- Mild	(28%; 7/25)	(66%; 2/3)	(33%; 1/3)	(17%; 1/6)	(0%; 0/1)	(50%; 1/2)	(33%; 2/6)
- Moderate	(4%; 1/25)	(0%; 0/3)	(0%; 0/3)	(0%; 0/6)	(0%; 0/1)	(0%; 0/2)	(0%; 0/6)
- Severe	(28%; 7/25)	(33%; 1/3)	(33%; 1/3)	(17%; 1/6)	(100%; 1/1)	(0%; 0/2)	(33%; 2/6)
Parietal lobe: (74%, 20/27)							
- None	(24%; 6/25)	(33%; 1/3)	(66%; 2/3)	(66%; 4/6)	(0%; 0/1)	(100%; 2/2)	(66%; 4/6)
- Mild	(20%; 5/25)	(66%; 2/3)	(33%; 1/3)	(0%; 0/6)	(100%; 1/1)	(0%; 0/2)	(0%; 0/6)
- Moderate	(16%; 4/25)	(0%; 0/3)	(0%; 0/3)	(33%; 2/6)	(0%; 0/1)	(0%; 0/2)	(0%; 0/6)
- Severe	(44%; 11/25)	(0%; 0/3)	(0%; 0/3)	(0%; 0/6)	(0%; 0/1)	(0%; 0/2)	(33%; 2/6)
Temporal lobe: (26%, 7/27)							
- None	(80%; 20/25)	(100%; 3/3)	(100%; 3/3)	(83%; 5/6)	(100%; 1/1)	(100%; 2/2)	(66%; 4/6)
- Mild	(24%; 6/25)	(0%; 0/3)	(0%; 0/3)	(17%; 1/6)	(0%; 0/1)	(0%; 0/2)	(33%; 2/6)
- Moderate	(0%; 0/25)	(0%; 0/3)	(0%; 0/3)	(0%; 0/6)	(0%; 0/1)	(0%; 0/2)	(0%; 0/6)
- Severe	(0%; 0/25)	(0%; 0/3)	(0%; 0/3)	(0%; 0/6)	(0%; 0/1)	(0%; 0/2)	(0%; 0/6)
Occipital lobe: (22%, 6/27)							
- None	(84%; 21/25)	(66%; 2/3)	(66%; 2/3)	(66%; 4/6)	(0%; 0/1)	(50%; 1/2)	(83%; 5/6)
- Mild	(12%; 3/25)	(33%; 1/3)	(0%; 0/3)	(0%; 0/6)	(100%; 1/1)	(0%; 0/2)	(0%; 0/6)
- Moderate	(4%; 1/25)	(0%; 0/3)	(33%; 1/3)	(17%; 1/6)	(0%; 0/1)	(50%; 1/2)	(17%; 1/6)
- Severe	(4%; 1/25)	(0%; 0/3)	(0%; 0/3)	(17%; 1/6)	(0%; 0/1)	(0%; 0/2)	(0%; 0/6)
Thalamus: (88%, 24/27)							
- None	(8%; 2/25)	(33%; 1/3)	(66%; 2/3)	(50%; 3/6)	(0%; 0/1)	(100%; 2/2)	(50%; 3/6)
- Mild	(20%; 5/25)	(66%; 2/3)	(0%; 0/3)	(0%; 0/6)	(100%; 1/1)	(0%; 0/2)	(17%; 1/6)
- Moderate	(20%; 5/25)	(0%; 0/3)	(0%; 0/3)	(17%; 1/6)	(0%; 0/1)	(0%; 0/2)	(0%; 0/6)
- Severe	(56%; 14/25)	(0%; 0/3)	(33%; 1/3)	(33%; 2/6)	(0%; 0/1)	(0%; 0/2)	(33%; 2/6)
Midbrain: (44%, 12/27)							
- None	(56%; 14/25)	(100%; 3/3)	(100%; 3/3)	(66%; 4/6)	(100%; 1/1)	(100%; 2/2)	(100%; 6/6)
- Mild	(24%; 6/25)	(0%; 0/3)	(0%; 0/3)	(0%; 0/6)	(0%; 0/1)	(0%; 0/2)	(0%; 0/6)
- Moderate	(12%; 3/25)	(0%; 0/3)	(0%; 0/3)	(17%; 1/6)	(0%; 0/1)	(0%; 0/2)	(0%; 0/6)
- Severe	(12%; 3/25)	(0%; 0/3)	(0%; 0/3)	(17%; 1/6)	(0%; 0/1)	(0%; 0/2)	(0%; 0/6)
Pons: (22%, 6/27)							
- None	(80%; 20/25)	(100%; 3/3)	(100%; 3/3)	(66%; 4/6)	(100%; 1/1)	(100%; 2/2)	(100%; 6/6)
- Mild	(12%; 3/25)	(0%; 0/3)	(0%; 0/3)	(17%; 1/6)	(0%; 0/1)	(0%; 0/2)	(0%; 0/6)
- Moderate	(8%; 2/25)	(0%; 0/3)	(0%; 0/3)	(0%; 0/6)	(0%; 0/1)	(0%; 0/2)	(0%; 0/6)
- Severe	(4%; 1/25)	(0%; 0/3)	(0%; 0/3)	(17%; 1/6)	(0%; 0/1)	(0%; 0/2)	(0%; 0/6)
Medulla: (11%, 3/27)							
- None	(92%; 23/25)	(100%; 3/3)	(100%; 3/3)	(66%; 4/6)	(100%; 1/1)	(100%; 2/2)	(100%; 6/6)
- Mild	(12%; 3/25)	(0%; 0/3)	(0%; 0/3)	(33%; 2/6)	(0%; 0/1)	(0%; 0/2)	(0%; 0/6)
- Moderate	(0%; 0/25)	(0%; 0/3)	(0%; 0/3)	(0%; 0/6)	(0%; 0/1)	(0%; 0/2)	(0%; 0/6)
- Severe	(0%; 0/25)	(0%; 0/3))	(0%; 0/3)	(0%; 0/6)	(0%; 0/1)	(0%; 0/2)	(0%; 0/6)
Cerebellum: (7%, 2/27)							
- None	(96%; 24/25)	(100%; 3/3)	(100%; 3/3)	(66%; 4/6)	(100%; 1/1)	(100%; 2/2)	(100%; 6/6)
- Mild	(4%; 1/25)	(0%; 0/3)	(0%; 0/3)	(17%; 1/6)	(0%; 0/1)	(0%; 0/2)	(0%; 0/6)
- Moderate	(0%; 0/25)	(0%; 0/3)	(0%; 0/3)	(0%; 0/6)	(0%; 0/1)	(0%; 0/2)	(0%; 0/6)
- Severe	(3%; 1/25)	(0%; 0/3)	(0%; 0/3)	(17%; 1/6)	(0%; 0/1)	(0%; 0/2)	(0%; 0/6)
Spinal cord: (4%, 1/27)							
- None	(96%; 24/25)	(100%; 3/3)	(100%; 3/3)	(100%; 6/6)	(100%; 1/1)	(100%; 2/2)	(100%; 6/6)
- Mild	(4%; 1/25)	(0%; 0/3)	(0%; 0/3)	(0%; 0/6)	(0%; 0/1)	(0%; 0/2)	(0%; 0/6)
- Moderate	(0%; 0/25)	(0%; 0/3)	(0%; 0/3)	(0%; 0/6)	(0%; 0/1)	(0%; 0/2)	(0%; 0/6)
- Severe	(0%; 0/25)	(0%; 0/3)	(0%; 0/3)	(0%; 0/6)	(%; 0/1)	(0%; 0/2)	(0%; 0/6)

Of the 21 animals that appeared to have been stunned effectively, which were then examined for gross brain pathology, all showed mild to severe damage to the thalamus, ten had damage to the midbrain, four also had damage to the pons, but none showed damage to the cerebellum and only one animal showed damage to the medulla. The majority (85%; 23/27) of all the animals examined were assessed as having some level of macroscopic damage to the thalamus, midbrain and brainstem structures (Table 11).

Of the six animals shot twice, only three were examined with gross brain pathology. The initial shots for all three of these animals deviated considerably from the HSA shooting position (between –40 and 80 mm caudal and –25 and 65 mm lateral). One shot completely missed the brain; there was some mild damage to the frontal lobe. With the second animal, the shot also missed the brain and resulted in no damage, however this animal was not shot again and showed only rhythmic breathing with no other signs of ineffective stunning. The third animal was found to have some damage to the occipital lobe, caused by a bone fragment from the first shot, but was not macroscopically damaged elsewhere (Table 12).

**Table 12.** Level of damage to cerebral lobes, thalamic and brainstem structures when horse brains were examined through macroscopic gross brain pathology and signs of consciousness observed post-stunning (total n = 27) at a commercial abattoir in Italy where horses were studied at slaughter

Post-stunbehaviour shown (%; n)^†^	Damage to cerebral lobes only	Damage to thalamic and brainstem structures	No macroscopic damage
Leg kicking (93%; 25/27)	12% (3/25)	92% (23/25)	4% (1/25)
Nystagmus (11%; 3/27)	33% (1/3)	66% (2/3)*	0% (0/3)
Eyeball rotation (11%; 3/27)	66% (2/3)	33% (1/3)*	0% (0/3)
Rhythmic breathing (22%; 6/27)	33% (2/6)	50% (3/6)	17% (1/6)
Spontaneous blinking (4%; 1/27)	0% (0/1)	100% (1/1)*	0% (0/1)
Failure to collapse (7%; 2/27)	100% (2/2)	0% (0/2)	0% (0/2)
Possible righting reflex (22%; 6/27)^†^	50% (3/6)	50% (3/6)	0% (0/6)

Animals were classified as ineffectively stunned after CBG shooting if they failed to collapse and/or rhythmic breathing was present and/or if at least two of the following parameters were present: positive corneal reflex, positive palpebral reflex, eyeball rotation and nystagmus

* Only thalamus damaged

† n = 5 also showed excessive leg kicking which complicated assessment of righting reflex.

When haemorrhage was assessed, 15% (4/27) of animals were deemed to have severe haemorrhage, 33% (9/27) had moderate haemorrhage, 44% (12/27) had mild haemorrhage and 7% (2/27) of animals were deemed to have no haemorrhage. Of those animals which were deemed to be ineffectively stunned, one displayed no haemorrhage with the rest displaying mild haemorrhage. There was no association between level of haemorrhage and stunning effectiveness (*P* = 0.098).

Based on Chi-squared tests, there were associations between stunning effectiveness and point of entry to the brain (*P* = 0.008), presence of damage to thalamic and brainstem structures (*P* < 0.001), damage to frontal lobe (*P* = 0.032) and to parietal lobe (*P* = 0.010). Univariate logistic regression results showed that the absence of damage to thalamic and brainstem structures meant that an animal was ten times more likely to show signs of ineffective stunning (*P* = 0.001; OR: 10.5, 95% CI: 2.5–44.8).

## Discussion

This study assessed equine welfare at slaughter in a commercial Italian abattoir, through exploring animal-based measures both ante mortem, at slaughter and post mortem. Crucially, it identified key factors that compromised animal welfare and the link between different stages and offers key recommendations to improve animal welfare throughout the slaughter process. It was hypothesised that equine welfare can be challenged throughout each stage of the slaughter process, and that associations would be found between factors including the level of stress experienced in the stunning box, stunning effectiveness and the level of brain damage caused by CBG. Floor condition and use of force by operators’ ante mortem appeared to be important factors affecting both stunning effectiveness and stress through increased slipping. EU recommendations (EC 2017) outline that floors should be kept clean and non-slip, but gives scarce equid-specific detail, and assessing compliance was not the aim of this study. More frequent cleaning of floors and development of non-slip designs, such as non-slip knurled metal with foot battens or wire mesh covers as used for horse transport vehicles (Nivelle *et al.* 2020; Zappaterra *et al.* 2022) could reduce slip risk (Grandin 2021) and subsequently improve stunning.

Operators shouting, as opposed to speaking calmly or remaining quiet, resulted in more force required for getting the horses into the stunning box and subsequently more animals slipping in the stunning box. This could be due to either operators getting frustrated with animals resisting, or the animals being shouted at consequently resisting movement, or these were more reactive or problematic animals, potentially due to background-related factors. However, based on the type of study (i.e. observational) and sample size, it was not possible to determine causal factors for this, although previous research has reported that negative human-animal interactions prior to slaughter increases stress behaviour (Hemsworth *et al.* 2011; Isbrandt *et al.* 2022).

More stress-related behaviours were seen in the stun box compared to the holding pen, including avoidance behaviour, ears back, blinking and sniffing the ground, suggesting that the stun box environment may cause increased stress. This might be due to the design (originally for cattle) not considering the increased height of horses, and so horses could see over the top of the box and into the bleeding and hoisting area, potentially explaining the high proportion of ‘orienting to stimulus’ behaviour seen at both stages. Witnessing the bleeding and processing of conspecifics has not been found to cause stress in pigs (*Sus scrofa*) and sheep (*Ovis aries*) (Anil *et al.* 1996, 1997). However, Sabiniewicz *et al.* (2023) found that horses displayed a potentially fearful response (assessed through backwards ear position) to the smell of horse blood, and Terlouw *et al.* (1998) reported that cattle showed increased air sniffing in response to the odours of stressed conspecifics, blood and dog faeces. Previous research has found that masking abattoir odours, sights and sounds from cattle (*Bos taurus*) and pigs can reduce stress (Grandin 2000; Lopez 2021) and Micera *et al.* (2010) found that the application of mentholated ointment to the nostrils of horses prior to slaughter reduced their adrenergic response. Therefore, installing walls to inhibit horses seeing or smelling the carcases of conspecifics post mortem might help to mitigate stress behaviours. However, other environmental factors could also have caused or exacerbated stress behaviour, such as noise, hosing down of equipment, changes in floor surface (and the prevalence of a poor floor condition due to faeces and blood from previously stunned horses, potentially explaining the prevalence of ‘sniffing ground’), or the entry to the stunning box itself (a darker, more confined space), alongside separation from conspecifics (Vermeulen *et al.* 2018; Fletcher *et al.* 2023) and the presence of researchers.

Whilst horses were moved as a group in the present study, they tended to be separated for slaughter when confined in the stunning box, although, on one occasion, the box was able to contain two animals for the purposes of co-slaughter, with the second animal shot immediately after the first. Anecdotally, these occasions were when the horses were unhandled and unrestrained, although tests were not conducted to determine whether a horse had been previously handled or not, or how this corresponded with age or origin, and there were insufficient numbers of unhandled horses to explore any impact of this. It would be useful to explore this further, with high numbers of unhandled horses arriving at abattoirs (Zappaterra *et al.* 2022). Previous research has found that unhandled horses can benefit from a conspecific presence at slaughter (Fletcher *et al.* 2023), especially considering the repercussions of this as regards shooting position, with the need for CBG to be shot at point-blank range.

The displaying of more stress-related behaviours such as avoidance and slipping in the stunning box increased the likelihood of those animals needing a second shot. This was potentially due to animals being harder to restrain, avoiding the operator, making them more challenging to stun in the appropriate position, and at point-blank, on the first attempt, causing a subsequent off-centre shot

Animals were shot with either a .22 or .25 penetrating CBG using 2.5 gr or 3.0 gr cartridges, respectively. It was a significant limitation of the study that the CBG/cartridge combination was not recorded for each animal. This made it impossible to draw conclusions on the effect that CBG/cartridge combination had on stunning effectiveness. Further research into the appropriate CBG/cartridge combination for different horse types is required, with currently no empirical evidence to provide guidance on this. A 2017 EU report advised that, in the absence of specific equid guidance, the same cartridge powerload as used for cattle (3.0–4.0 gr) should be used for stunning equids (EC 2017). However, equids differ considerably from cattle as regards morphology and behaviour, both compared to other taxa and between breeds/types, with some breeds of horses, such as purpose-bred meat or draught horses included in this sample, weighing in excess of half to three-quarters of a tonne (Lorenzo *et al.* 2014; Razmaitė *et al.* 2021). Therefore, adapting abattoir processes and equipment to accommodate these differences and conducting further research to determine appropriate CBG powerload could subsequently improve welfare, particularly stunning effectiveness.

Twenty-two percent of animals (n = 14) in this study showed signs of ineffective stunning (rhythmic breathing and/or eye reflexes), although only six of these displayed rhythmic breathing without any additional signs. Five of these animals also showed excessive leg kicking which could have caused complications or misinterpretation in assessment of rhythmic ribcage movement, and so the numbers of animals showing rhythmic breathing, particularly where other signs were absent, should be interpreted with caution. Furthermore, righting reflex was termed ‘possible’, with animals constricted by the stunning box and/or displaying excessive leg kicking. Therefore, it was difficult to interpret whether they were indeed attempting to regain posture, or if it was leg kicking or the position they fell in, hence this parameter was not included in classification as ineffectively stunned. The criteria incorporating definition as ineffectively stunned, rhythmic breathing and/or eye reflexes, may not necessarily indicate immediately compromised welfare but suggests that these animals might have the potential to recover consciousness if prompt exsanguination does not occur. They should therefore be checked for other potential signs until the end of the bleeding period (Terlouw *et al.* 2016) and, if appropriate, operators should intervene with a second shot (Gregory *et al.* 2007; Gibson *et al.* 2012, 2015b). Of those 22% (n = 14) animals showing signs of ineffective stunning, only five were shot twice (note n = 1/6 of the horses shot twice had no signs of ineffective stunning), suggesting that abattoir operators did not deem it necessary to shoot a second time, or potentially either misinterpreted signs or did not recognise more subtle signs of possible consciousness.

It is unclear what signs led to the decision to take a second shot, but factors could have included the processing line speed, horse type (primarily whether horses were accustomed to handling or were feral, although this was not formally assessed) and the abattoir design (whereby the shooting operator could not easily see into the bleeding area when the horse was ejected from the stunning box). Additionally, there is the potential that operators may have been cautious to shoot a second time due to the requirement to record multiple shots under European slaughter regulations (EC 1099/2009 2009), a practice which aims to detect non-compliance, but which has been previously found during audits to deter European abattoir operators from a second shot attempt (Paolucci *et al.* 2015). However, these factors were not examined in the current project. Shot placement position was not recorded at the time of shooting, however shot order was determined based on position and gross pathology.

All but one animal shot twice showed signs of rhythmic breathing. Whilst rhythmic breathing, in isolation, is not indicative of ineffective stunning, it allows oxygenated blood to be delivered to the brain, pre-exsanguination, that may then support the maintenance, or recovery, of brain function and subsequent consciousness (Borzuta *et al.* 2019). Theoretically, correct penetrating or non-penetrating CBG stunning should cause an immediate cessation of respiration (Comin *et al.* 2023) but this relies on focal and/or diffuse injury to the medulla and pons, responsible for unconscious respiratory regulation (Gregory *et al.* 2009; Schottelkotte & Crone 2022). This corresponds with this study’s findings that no animals showing rhythmic respiration, which were then examined for gross brain pathology, were found to have damage to the pons or medulla. However, one of these animals had moderate damage to the midbrain. Animals that have damage to the midbrain might demonstrate agonal or spasmodic gasping, but this is easily discriminatory from rhythmic breathing, being more intermittent (Grist *et al.* 2018) and is not functional respiratory activity (Gregory *et al.* 2009). Overall, absence of damage to thalamic, midbrain and brainstem structures meant that an animal was more likely to show signs of ineffective stunning. This is in line with research in alpacas (*Vicugna pacos*), which found that appropriate positioning of the penetrating CBG to maximise the probability of damaging the thalamus and brainstem was especially important to ensure irrecoverable complete insensibility (Gibson *et al.* 2015b).

For animals in the study that were successfully stunned but did not have damage to critical brain structures, this could potentially be due to microscopic damage (Al-Sarraj 2016) and/or concussive contrecoup/acceleration/deceleration injury separate to the physical trauma from the bolt. This is seen during non-penetrating CBG (NPCB), which operates through the transference of force (kinetic energy) from the rapidly moving bolt to the skull and brain to concuss the animal (Oliveira *et al.* 2018). However, physical damage to brain tissue structures is generally less extensive and severe with NPCB compared to penetrating CBG, which increases the potential for ineffective stunning or recovery from concussion (Oliveira *et al.* 2018; Sussman *et al.* 2018). One animal, where the shot missed the brain and did not have damage to brainstem structures, was found to have some very mild damage to the frontal lobe, which could have been caused by contrecoup forces, extraction artefact, or from the second shot the animal received. Generally, the variability in damage caused to the brains of animals in this study highlights the importance of ensuring equipment is appropriate for the species.

Any delays between the stun and exsanguination further increases the potential of ineffectively stunned animals recovering breathing function and regaining consciousness. The median time between stunning and bleeding was 69.5 s, with the longest time at 130 s, likely impacted by researcher examination of animals, post-stun. The HSA recommends when using penetrating CBG a maximum stun to stick interval of 60 s (HSA 2013), whilst the WOAH and a report by the European Commission recommend 20 and 25 s, respectively (EC 2017; WOAH 2021). However, whether this is practical and realistic under commercial slaughter conditions, where animals require mechanical hoisting post-stun and prior to bleeding, needs further exploration. Werner and Gallo (2008) reported that 57.2% of horses sampled showed return to sensibility when there was a delay of up to 4 min between stunning and bleeding. It is worth noting that, in the present study, researcher assessment contributed to the delay between stunning and bleeding, as the examination of behavioural/brainstem indices was performed immediately post-stun, taking at least 10 s. The delay was also likely due to situations where the operator struggled to shackle the animal post-stun, due to excessive clonic convulsions and movement, such as leg kicking, displayed by 90% of animals, which presented a safety risk and occasionally prevented examination of the animal by the researcher. This may have also prevented a second shot being given promptly in some cases, or the need for one being identified by operators. The high prevalence of leg kicking post-stunning is a potentially reassuring indicator of effectiveness, with cattle and sheep showing involuntary paddling movements for up to a minute demonstrating other signs consistent with an effective stun (Gibson *et al.* 2012; Terlouw *et al.* 2016), and the paddling likely to be an involuntary movement independent of consciousness. Similarly, brain-dead, artificially ventilated humans have shown certain limb movements believed to be related to residual nerve activity in the spinal cord (Laureys 2005).

Grist *et al.* (2019) reported that post-stun reflexes were not associated with deviation from ideal shot position in cattle. However, Gibson *et al.* (2015b) found that this was not the case in alpacas and deviation was associated with reflexes. This contrast in findings may be due to differences in CBG and powerload combination, with powerload fill weight impacting velocity and kinetic energy (Grist *et al.* 2019) or due to differences in species morphology, such as skull thickness and the size of the brain in relation to the skull varying amongst species and breeds. Horses have been found to have differently shaped brains to *Cetartiodactyla* such as cattle and alpacas (Cozzi *et al.* 2014). It is also important to note that, in the present study, the deviation was measured on skinned heads, whereby the lack of ears and skin presented challenges with determining the HSA shooting position (20 mm above the intersection between the middle of each eye to the base of the opposite ear, with the muzzle of the firearm slightly tilted to direct the shot through the cerebral cortex towards the brain stem; HSA 2013). The findings of this study cautiously support this position, although further research is needed into positioning for CBG stunning in horses.

Achieving a CBG shot at exactly the HSA position is extremely difficult to achieve. With 43% of the sample shot at less than 10 mm away from this position, this suggests a high degree of accuracy by the operator, with 80% of animals shot within 10 mm from this position seemingly stunned effectively. However, more than half were shot at more than 10 mm deviation and 39% at more than 20 mm deviation, with the maximum deviation 80 mm caudally and most shots to the right of the HSA position. Shooting accuracy and effectiveness were possibly influenced by the position and laterality of the operator (in the present study, stood above the stunning box and right-handed), along with the design of the stunning box, lack of head restrainers and the presence and position of the researcher observing the process of stunning. This is still an improvement compared with some studies of cattle, where almost 80% of animals examined were found to have been shot more than 20 mm from the recommended shooting position (Vecerek *et al.* 2020). However, this did not predict stunning efficiency. This is in line with the present study where ineffective stunning was determined in only 31% of animals with more than 20 mm deviation and two animals shot within 10 mm showed signs of ineffective stunning. However, these latter two animals only showed rhythmic respiration and, when examined post mortem, there was no damage to the medulla, which is responsible for regulating respiratory activity (Gregory *et al.* 2009; Schottelkotte & Crone 2022). This raises the question of whether there is perhaps a greater margin of error from the HSA suggested position, as long as shots are at a sufficient depth and angle to target critical brain structures. These results highlight that further empirical research is required to determine if the HSA position is appropriate.

Shot depth and delivered kinetic energy (function of velocity and mass) are associated with CBG performance (Gibson *et al.* 2014; Grist *et al.* 2019). Poorly maintained CBGs can underperform and malfunction (Gibson *et al.* 2014; Grist *et al.* 2019) and should be routinely checked and maintained, particularly when repeat firing occurs in a session, to prevent carbon build-up and ensure optimal performance (Gibson *et al.* 2014). Lower grain cartridges have been found to vary more in weight, volume and velocity than higher grain cartridges (Gibson *et al.* 2014; Grist *et al.* 2019). This can increase the risk of a miss-stun or poor performance, although this variation is unlikely to be detected by slaughter operators. However, a shallow depth of concussion in cattle has been found to be associated with soft-sounding (lower decibel) shots using higher-grain (4.5 gr) cartridges (Gregory *et al.* 2007).

This study is potentially biased by abattoir personnel being aware of the presence of the researchers, which could have distracted or potentially resulted in them adapting their usual behaviour, positively or negatively. The presence of additional humans might also have increased stress in animals throughout the processing line. The ability to video record would have allowed retrospective checking of data, alongside recording time between shots and signs of consciousness during bleeding more accurately, however this is often not permitted in the abattoir environment. In addition, the study was limited by the sample size, which differed at each stage due to missing data, the binary behavioural sampling method (one-zero) which did not record duration or frequency of behaviours, and the difference in time windows of observations. This prohibited the analysis of any associations between post-stun behaviours and gross brain pathology and so, importantly for future work, these signs should be triangulated to strengthen guidance surrounding appropriate CBG positioning. Measuring deviation from suggested shot position on skinned heads presented difficulties, whilst histopathology and microscopic brain analysis could have provided some additional detail regarding the level of damage necessary for effective stunning. However, these limitations are to be expected from a field-based, convenience-sample study and the findings are not only novel, but also valuable in paving the way for the implementation of a standardised protocol for assessing equid welfare at slaughter.

## Animal welfare implications and conclusion

This study is the first to investigate the potential association between ante mortem behaviour, stun effectiveness and pathological indicators in horses at slaughter. Relationships were found between stress-related behaviours such as slipping and avoidance behaviour, negative human-animal interactions such as personnel vocalisations and use of force, and ineffective stunning, including the requirement for repeat shooting. Incremental species-specific improvements should also be introduced to abattoir design, such as ensuring flooring condition and stunning box design are appropriate for equids, to minimise slip risk and mitigate stress. Abattoir personnel should be suitably knowledgeable regarding species-appropriate handling, appropriate gun positioning to target critical brain structures, and signs of either an insufficient or shallow depth of concussion that would require a secondary intervention. This could then improve effectiveness, operator safety and animal welfare.

## Supporting information

Fletcher et al. supplementary material 1 Fletcher et al. supplementary material

Fletcher et al. supplementary material 2 Fletcher et al. supplementary material

## Supplementary material

The supplementary material for this article can be found at http://doi.org/10.1017/awf.2024.70.
