# Assessment of ante mortem welfare indicators and the pathophysiology of captive-bolt trauma in equids at slaughter - CORRIGENDUM

**DOI:** 10.1017/awf.2025.14

**Published:** 2025-03-12

**Authors:** Katharine A Fletcher, Barbara Padalino, Martina Felici, Daniele Bigi, Georgina Limon-Vega, Andrew Grist, Troy J Gibson

**Keywords:** Animal welfare, captive-bolt gun, Equus caballus, killing, post mortem, veterinary pathology

In the paper *Fletcher KA, Padalino B, Felici M, Bigi D, Limon-Vega G, Grist A and Gibson TJ (2024)*, horses were evaluated at a commercial Italian abattoir. Ante mortem assessment using animal-based measures was conducted on 62 horses and 23 horses were also analysed post mortem, including assessment of heads for deviation of Captive Bolt Gun (CBG) stunning position from that suggested by the Humane Slaughter Association (HSA).

However, there was a mistake in the original calculations performed for this position, with the suggested HSA position taken as that recommended for free bullet firearm shooting rather than CBG stunning. For free bullet shooting, the HSA suggests a frontal shooting position of 20 mm above the intersection of lines drawn from the middle of each eye to the base of the opposite ear with the muzzle of the firearm angled towards the neck (HSA 2013). However, for CBG stunning, the HSA suggests a frontal shooting position of 10 mm above the intersection of lines drawn from the middle of each eye to the base of the opposite ear with the muzzle of the firearm angled towards the neck (HSA 2013).

When we realised that the calculations for deviation from suggested position were incorrect, we immediately recalculated all associated results. Following this, Figure 2, comparing deviation from the HSA suggested position (for CBG stunning) with either signs of an ineffective stun or whether a second shot was given had to be revised (see below):

**Figure 2.**
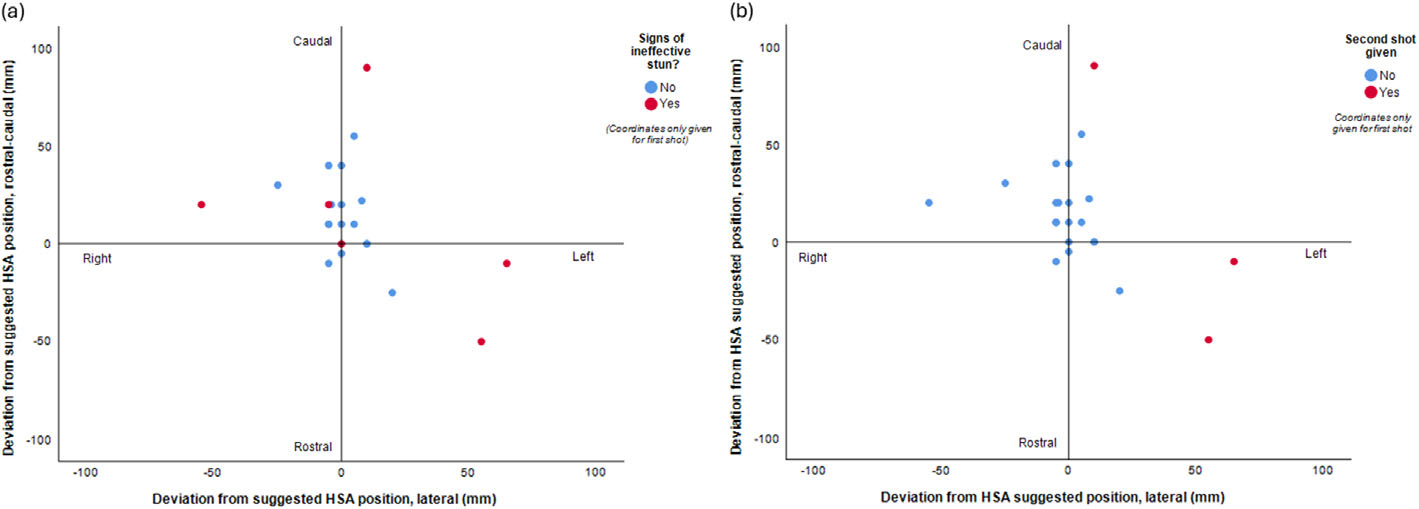
Scatterplot showing deviation from the suggested Humane Slaughter Association’s (HSA) position for captive bolt shooting of horses (- is left from operator’s perspective and rostral of midline), showing (a) where animals showed signs of effective or ineffective stunning and (b) if a second shot was given (n = 23).

In addition, the proportions deviating from this suggested position had to be revised. Originally, 43% (10/23) animals were determined to have no (≤ 10 mm sagittal and/or lateral) deviation from the HSA position; this has now changed to 9% (2/23). Of the remaining animals, 90% (19/21) and 43% (9/21) had ≥ 10 mm rostral-caudal or lateral deviations from the suggested shooting position respectively.

Thirty percent (7/23) were shot at between 10 and 20 mm deviation (rostral-caudal and/or lateral) and 61% (14/23) at more than 21 mm deviation. The maximum deviation was 90 mm caudally and 65% (15/23) shots to the left of the animal’s midline. Of those animals examined for gross brain pathology, 26% (6/23) showed signs of ineffective stunning, of which 83% (5/6) had rostral-caudal deviation of ≥ 10 mm and 67% (4/6) had rostral-caudal deviation of ≥ 20 mm.

We also recognised that including positive and negative scores in our measurements (with ‘minus’ indicating the shot was rostral or to the right of the suggested position) affected the descriptive statistics in terms of calculating the mean and standard deviation. These were therefore revised to determine that the mean sagittal deviation was 22.0 ± 20.8 (previously was stated as being 3.4 ± 7.2), with a range of –50 to 90 (previously –60 to 80), and the mean lateral deviation was 13.8 ± 18.5 (previously was stated as being 3.6 ± 22.9). Apart from these changes, further corrections were unnecessary, since all other results and conclusions were essentially the same. These errors should have been picked up by us prior to publishing the article, and we regret any inconvenience caused by these oversights.
