# Supplementary material for: Assessment of ante mortem welfare indicators and the pathophysiology of captive-bolt trauma in equids at slaughter
Source: Anim Welf. 2025 Jan 3;33:e65. doi: 10.1017/awf.2024.70 (PMC11704570; doi:10.1017/awf.2024.70)
Supplement: Fletcher et al. supplementary material 1 — Fletcher et al. supplementary material [file S0962728624000708sup001.pdf]

HOLDING PEN	Date: ....
**Animal ID**	

**Species**	Horse ☐ *(Sports/TB/dolicomorfe ☐ Cob/native/mesomorfe ☐ Purpose-bred meat horse/draft/brachimorfe) ☐)* Donkey ☐ Mule ☐
**Body Condition Score**	☐ 0 - Emaciated ; 1 - Underweight ☐; 2 - Moderate ☐; 3 - Healthy ☐ ; 4 - Overweight ☐ ; 5 - Obese ☐ ; Unable to assess ☐

**Ocular discharge**	Present ☐ Absent ☐ Not observed ☐	*Additional comments:*
**Nasal discharge**	Present ☐ Absent ☐ Not observed ☐	
**Abnormal respiration**	Present ☐ Absent ☐ Not observed ☐	
**Signs of disease/ infection**	Present ☐ Absent ☐ Not observed ☐	
**Skin/coat issues**	Present ☐ Absent ☐ Not observed ☐	
**Lesions/wounds**	Present ☐ Absent ☐ Not observed ☐	
**Scars/swellings**	Present ☐ Absent ☐ Not observed ☐	
**Foot/limb abnormalities**	Present ☐ Absent ☐ Not observed ☐	
**Lameness**	Present ☐ Absent ☐ Not observed ☐	Details:
**Slips/falls**	Present ☐ Absent ☐ Not observed ☐	

**Ethogram (conspicuous behaviour sampling, 1-0)**	**Total time observed (min/secs):** ....

**State Behaviours**	☑	**Head Behaviours**	☑	**Eye Behaviours**	☑	**Locomotive Behaviours**	☑
Sweating		Orient to bleeding area		Sclera visible		Kick/kick threat	
Calm/alert		Ears back		High blink rate		Stomping	
Hyper-responsive		Head shaking		Yawning		Pawing	
Dull/depressed		Pull back on halter		**Mouth behaviours**	☑	Weight shifting	
Restlessness		Turn head/avoidant		Lick/chew		Trembling/shivering	
Score: ☑ if present, Blank if not present 'N/O' not able to observe		Head lowered		Vocalisation (call/scream)		Urination	
		Sniffing ground		Snort		Defecation	
				Bite/bite threat		Rear	
						Rapid breathing/↑resp	

**Other behaviours of note**		
**External factors affecting behaviour**	*(e.g. blood splatters, water, reaction to visibility of carcasses, noises, etc)*	
**Entry to kill pen**	• Willingly ☐<br>• Needs encouragement/mild pressure ☐<br>• Requires force/significant pressure ☐	Additional comments:
**Equipment used (device)**	Stick/whip ☐ electrical ☐ none ☐ other ☐................<br>Type (e.g.plastic/wood/metal).................................	
**Equipment used (manner)**	• Not applicable/none ☐<br>• Gentle (soft/light tap <u>OR</u> <5x) ☐<br>• Intense (Strong tap/Arm raised but no damage <u>OR</u> >5-<10x) ☐<br>• Rough (Arm raised >shoulder height/excessive force <u>OR</u> >10x) ☐	
**Response to operator**	Avoidant/nervous☐ agonistic/aggressive☐ friendly/affiliative☐<br>calm/alert☐ apathetic/depressed☐ not observed☐	
**Personnel vocalisations**	• Speaking (speaks or whistles softly/quietly) ☐<br>• Shouting (speaks or shouts harshly/loudly) ☐<br>• Rattling/slamming (makes noise/claps hands/slamming wall) ☐<br>• None/nothing of note ☐<br>• Not observed ☐	
**Personnel attitude**	• Positive (e.g. talking quietly, petting, touching) ☐<br>• Neutral (e.g. dominant talking, gentle touch of stick/hand) ☐<br>• Negative (e.g. talks/shouts impatiently, forceful use of stick/hand) ☐<br>• Nothing of note ☐ Not observed ☐	

SLAUGHTER	ID:	Date: ……………………………………………………
**Head profile**	Concave ☐ Convex ☐ Straight ☐	Additional comments:
**Level of restraint**	• 0= No restraint/halter (loose) ☐<br>• 1=mild (halter&lead but no pulling/tension) ☐<br>• 2=moderate (halter/lead,minimal pressure/tension) ☐<br>• 3=severe (halter/lead, significant pressure/tension) ☐	
**Equipment used (device)**	Stick/whip ☐ electrical ☐ none ☐ other ☐………………….<br>Type (e.g. plastic/wood/metal)…………………………………….	
**Equipment used (manner)**	• Not applicable/none ☐<br>• Gentle (soft/light tap <u>OR</u> <5x) ☐<br>• Intense (Strong tap/Arm raised but no damage <u>OR</u> >5-<10x) ☐<br>• Rough (Arm raised >shoulder height/excessive force <u>OR</u> >10x) ☐	
**Response to personnel**	Avoidant/nervous ☐ agonistic/aggressive ☐ friendly/affiliative ☐<br>calm/alert ☐ apathetic/depressed ☐ not observed ☐	
**Personnel vocalisations**	• Speaking (speaks or whistles softly/quietly) ☐<br>• Shouting (speaks or shouts harshly/loudly) ☐<br>• Rattle/slam (claps hands, slams wall etc) ☐<br>• Nothing of note ☐ Not observed ☐	
**Personnel attitude**	• Positive (e.g. talking quietly, petting, touching) ☐<br>• Neutral (e.g. dominant talking, gentle touch of stick/hand) ☐<br>• Negative (e.g. talks/shouts impatiently, forceful use of stick/hand) ☐<br>• Nothing of note ☐ Not observed ☐	
**Slips**<br>**Falls**	Present ☐ Absent ☐ Number: ………..<br>Present ☐ Absent ☐ Number: ………..	
**Floor surface**	Poor ☐ Moderate ☐ Good ☐ Not observed ☐<br>Details:	

**Ethogram (continuous sampling, conspicuous/focal behaviour)**				**Time in kill pen: ………………(min/secs)**			
**State Behaviours**	☑	**Head Behaviours**	☑	**Eye Behaviours**	☑	**Locomotive Behaviours**	☑
Sweating		Orient to bleeding area		Sclera visible		Kick/kick threat	
Calm/alert		Tries to turn in box /back-up		High blink rate		Stomping	
Hyper-responsive		Ears back		Frequent yawning		Pawing	
Dull/depressed		Head shaking		**Mouth behaviours**	☑	Weight shifting	
Restlessness		Pull back on halter		Lick/chew		Trembling/shivering	
Score: ☑ if present, Blank if not present ‘N/O’ not able to observe		Turn head/avoidant		Vocalisation (call/scream)		Urination	
		Head lowered		Snort		Defecation	
		Sniffing ground		Bite/bite threat		Rear	
						Rapid breathing/↑resp	
**Other behaviours of note**							

**Primary method of slaughter**	Captive Bolt ☐<br>Details:………………………………………………	**If 2nd shot:** (Total no. of shots)<br>……………
**Shot aiming position**	Frontal ☐ Side ☐ Rear ☐ Not observed ☐	Frontal ☐ Side ☐ Rear ☐ Not observed ☐
**Shot distance**	Not observed ☐ Point blank (touching head) ☐<br>Not point blank (details):<br>……………………………………………………………………	Not observed ☐ Point blank (touching head) ☐<br>Not point blank (details):<br>……………………………………………………………………
**Immediate collapse**	Present ☐ Absent ☐<br>Could not assess ☐	Present ☐ Absent ☐<br>Could not assess ☐
**Any other signs of insensibility / sensibility observed**		

BLEEDING/HOISTING	ID:	Date: ………………………………………………………………	
**Signs of shooting effectiveness (sensibility/insensibility)**	**1st shot**	**2nd shot**	**(if 2+ shots, comment any additional info)**
**Immediate collapse**	Present ☐<br>Absent ☐<br>Could not assess ☐	Present ☐<br>Absent ☐<br>Could not assess ☐	Additional comments:
**Leg kicking/convulsions**	Present ☐<br>Absent ☐<br>Could not assess ☐	Present ☐<br>Absent ☐<br>Could not assess ☐	
**Gasping**	Present ☐<br>Absent ☐<br>Could not assess ☐	Present ☐<br>Absent ☐<br>Could not assess ☐	
**Nystagmus**	Present ☐<br>Absent ☐<br>Could not assess ☐	Present ☐<br>Absent ☐<br>Could not assess ☐	
**Eyeball rotation**	Present ☐<br>Absent ☐<br>Could not assess ☐	Present ☐<br>Absent ☐<br>Could not assess ☐	
**Rhythmic respiration**	Present ☐<br>Absent ☐<br>Could not assess ☐	Present ☐<br>Absent ☐<br>Could not assess ☐	
**Corneal reflex**	Present ☐<br>Absent ☐<br>Could not assess ☐	Present ☐<br>Absent ☐<br>Could not assess ☐	
**Palpebral reflex**	Present ☐<br>Absent ☐<br>Could not assess ☐	Present ☐<br>Absent ☐<br>Could not assess ☐	
**Blinking**	Present ☐<br>Absent ☐<br>Could not assess ☐	Present ☐<br>Absent ☐<br>Could not assess ☐	
**Muscle spasms**	Present ☐<br>Absent ☐<br>Could not assess ☐	Present ☐<br>Absent ☐<br>Could not assess ☐	
**Painful response to ear pinch**	Present ☐<br>Absent ☐<br>Could not assess ☐	Present ☐<br>Absent ☐<br>Could not assess ☐	
**Painful response to nostril pinch**	Present ☐<br>Absent ☐<br>Could not assess ☐	Present ☐<br>Absent ☐<br>Could not assess ☐	
**Vocalisations**	Present ☐<br>Absent ☐<br>Could not assess ☐	Present ☐<br>Absent ☐<br>Could not assess ☐	
**Righting reflex**	Present ☐<br>Absent ☐<br>Could not assess ☐	Present ☐<br>Absent ☐<br>Could not assess ☐	
**Painful response to knife/cut**	Present ☐<br>Absent ☐<br>Could not assess ☐	Present ☐<br>Absent ☐<br>Could not assess ☐	
**Defecation**	Present ☐<br>Absent ☐<br>Could not assess ☐	Present ☐<br>Absent ☐<br>Could not assess ☐	
**Urination**	Present ☐<br>Absent ☐<br>Could not assess ☐	Present ☐<br>Absent ☐<br>Could not assess ☐	
**Ventral neck incision**	**No. of cuts**		
	**Time stun to stick**	1st cut…………… (seconds) 2nd cut…………… (seconds)	
	**Arteries cut/severed**	Could not assess ☐ Facial artery ☐<br>Right Carotid ☐ Left Carotid ☐ Right Jugular ☐ Left Jugular ☐<br>Total severance of brachiocephalic trunk ☐	

POST-MORTEM	ID:	Date: …………………………………………………………
**Point of entry**	Frontal lobe ☐ Parietal lobe ☐ Temporal lobe ☐ Occipital lobe ☐ Cerebellum ☐ Missed brain ☐ Other……………………………… Could not assess ☐	Additional comments:
**Extrusion of tissue from bolt cavity**	Present ☐ absent ☐ N/A ☐ Could not assess ☐	
**Cranial fractures**	Present ☐, absent ☐; depressed ☐ radiating ☐ Other……………. Measure if necessary ………….. Could not assess ☐	
**Skull thickness** (at impact site)	……..(mm) Could not assess ☐	
**Macroscopic subdural haemorrhage to intercranial space**	Left hemisphere …….(%) Right hemisphere …….(%) Total ………..(%) Could not assess ☐	
**Haemorrhage in wound cavity**	Present = Y, absent = N; Details……….. Could not assess ☐	
**Bullet/bolt trajectory**	Could not assess ☐	
**Tissue displacement**	Present = Y, absent = N; Position…………………… Could not assess ☐	
**Cerebellum coning**	Present = Y, absent = N Could not assess ☐	
**Bone fragmentation**	Present = Y, absent = N; Number…… Position……………………….. Could not assess ☐	
**Do bone and bullet fragments have a trajectory different to main path of bolt/bullet**	Yes/No, details: Could not assess ☐	
**Cavitation** (inner surface of cranial vault)	Present = Y, absent = N; Position…………………………………. Could not assess ☐	
**Petechiae**	Pons ☐ Thalamus ☐ Cerebellum ☐ Midbrain ☐ Frontal lobe ☐ Proprietal lobe ☐ Occipital lobe ☐ Temporal lobe ☐ Medella ☐ Reticular formation ☐ **Not observed ☐** Could not assess ☐	
**Macroscopic subdural tissue damage to brain regions**	None ☐ Mild ☐ Moderate ☐ Severe ☐ Not seen ☐ *(None = 0%; Mild <20%, Moderate=21%-49%; Severe >50%)* Could not assess ☐	
**Frontal lobe:**	None ☐ Mild ☐ Moderate ☐ Severe ☐ Could not assess ☐	
**Parietal lobe:**	None ☐ Mild ☐ Moderate ☐ Severe ☐ Could not assess ☐	
**Occipital lobe:**	None ☐ Mild ☐ Moderate ☐ Severe ☐ Could not assess ☐	
**Temporal lobe:**	None ☐ Mild ☐ Moderate ☐ Severe ☐ Could not assess ☐	
**Midbrain:**	None ☐ Mild ☐ Moderate ☐ Severe ☐ Could not assess ☐	
**Pons:**	None ☐ Mild ☐ Moderate ☐ Severe ☐ Could not assess ☐	
**Thalamus:**	None ☐ Mild ☐ Moderate ☐ Severe ☐ Could not assess ☐	
**Cerebellum:**	None ☐ Mild ☐ Moderate ☐ Severe ☐ Could not assess ☐	
**Medella:**	None ☐ Mild ☐ Moderate ☐ Severe ☐ Could not assess ☐	
**Spinal chord:**	None ☐ Mild ☐ Moderate ☐ Severe ☐ Could not assess ☐	
**Macroscopic haemorrhage to brain regions**	Ventral surface☐ 4th ventricle☐ 3rd ventricle☐ Lateral ventricles ☐ Cerebral aqueduct ☐ Pons ☐ Thalamus ☐ Cerebellum ☐ Midbrain ☐ Frontal lobe ☐ Parietal lobe ☐ Occipital lobe ☐ Temporal lobe ☐ Medella ☐ Could not assess ☐	
**Overall haemorrhage**	None ☐ Mild ☐ Moderate ☐ Severe ☐ Not seen ☐	

Date: ……………………………………………….

	Deviation from ideal position (sagittal)	Deviation from ideal position (Lateral)	Length of head	Distance shot to tip of nose	Width of head	Distance between eyes	Angle of shot (transverse)	Angle of shot (sagittal)
	Calculated using acetate with ideal position marked 2cm above where lines cross from eye to ear		(poll to tip of nasal bone)	(shot-position to tip of nasal bone)	(between widest point of eyes)	(inner corner of each eye)	Calculated using protractor with angle reader in shot hole	
ID	(mm)	(mm)	(mm)	(mm)	(mm)	(mm)	(Degrees °)	(Degrees °)

General lairage information:

Date:		
**Indicator**	**Score**	**Description/criteria**
**Air circulation/ventilation**	Poor ☐ Moderate ☐ Good ☐ Not observed ☐<br>Wind speed: ………………. (m/sec)	Poor=none, mod=some but not reaching animal, good=well ventilated, clear unpolluted air
**Temperature/humidity**	……… (°C) Unable to measure ☐	
**Noise levels**	……… (decibels) Unable to measure ☐	
**Cleanliness of pen/hazards**	Poor ☐ Moderate ☐ Good ☐ Not observed ☐	Poor=multiple faeces and severe hazards, moderate=minor hazards and some faeces, good=minimal faeces and minimal/none hazards
**Food**	Present ☐ Absent ☐	
**Water**	Present ☐ Absent ☐	
**Bedding**	Present ☐ Absent ☐	
**Shade/shelter**	Present ☐ Absent ☐	
**Vision/lighting**	Poor ☐ Moderate ☐ Good ☐ Not observed ☐	Poor=none, mod=some but either too bright/artificial or limited, good=well lit with some natural light
**Floor surface/slip risk**	Poor ☐ Moderate ☐ Good ☐ Not observed ☐	Poor=trip hazards and/or significant slip risks e.g. faeces/wetness, mod=flooring not suitable due to level of risk from slips/falls from wetness/minor hazards, good=absence of hazards or wetness/faeces, unobstructed dry/level flooring
**Distance from lairage to holding pen**		
**Size of lairage pen**		

**Movement** (from lairage to holding pen)		**Date/Time of assessment: ……………..**
	**Animal ID/details + position/location in lairage**	
**Species**	Horse ☐ *(Sports/TB/dolicomorfe ☐ Cob/native/mesomorfe ☐ Purpose-bred meat horse/draft/brachimorfe) ☐)* Donkey ☐ Mule ☐	
**Level of restraint**	0= No restraint/halter (loose) ☐<br>1=mild (restraint with halter&lead but no pulling/tension) ☐<br>2=moderate (restraint with halter/lead minimal pressure/tension) ☐<br>3=severe (restraint with halter/lead with significant pressure/tension) ☐	Additional comments:
**Equipment used (device)**	Stick/goad/whip ☐ electrical ☐ none ☐ other ☐ …………………..	
**Equipment used (manner)**	Gentle (soft/<5x) ☐<br>Intense (stronger than before without damaging, >5-<10x) ☐<br>Rough (>10x, excessive force, damaging) ☐<br>*(adapted from Huertas et al, 2018)* Not applicable/none ☐	
**Response to personnel/operator**	Avoidant/nervous ☐ agonistic/aggressive ☐ friendly/affiliative ☐<br>calm/alert ☐ apathetic/depressed ☐ not observed ☐	
**Personnel vocalisations**	Speaking (speaks or whistles softly/quietly) ☐<br>Shouting (speaks or shouts harshly/loudly) ☐<br>Rattling/slamming (makes noise/claps hands/slamming wall) ☐<br>None/nothing of note ☐ Not observed ☐	
**Personnel attitude**	Positive (e.g. talking quietly, petting, touching) ☐<br>Neutral (e.g. dominant talking, gentle touch of stick/hand) ☐<br>Negative (e.g. talks/shouts impatiently, forceful use of stick/hand) ☐<br>Nothing of note ☐ Not observed ☐	
**Slips**<br>**Falls**	Present ☐ Absent ☐ Number: ……<br>Present ☐Absent ☐ Number: …..	
**Lameness**	*Weight bearing on all 4 = no; holding up leg = yes*	Yes ☐ No ☐ Not observed ☐

Date of assessment:

ID (order)	Microchip	Time/date of animal's arrival at abattoir	Year of Birth	Origin	Any other details

Appendices

(B): Ethogram for response to operator or to conspecifics

**Categorisation**	**Definition**	**Reference**
Avoidant/nervous	Moves or attempts to move away/turns head away	Burn et al, 2010
Agonistic/aggressive	Attempts to bite, rear, kick or strike with foreleg; ears held back or flattened	
Friendly/affiliative	Turns head towards/ears forward	
Calm/alert	Responding to surroundings e.g. ears moving and often forward, eyes open, feet may be moving, tail swishing, head up unless sniffing or eating	
Apathetic/depressed	Passive response to surroundings e.g. small ear movements, some tail swishing, feet may be moving, eyes may be half-closed, head may be lowered/unresponsive to surroundings, e.g. ears still and lowered, eyes closed or half-closed, no tail swishing or foot movement, head lowered	
