# Supplementary material for: Assessment of ante mortem welfare indicators and the pathophysiology of captive-bolt trauma in equids at slaughter
Source: Anim Welf. 2025 Jan 3;33:e65. doi: 10.1017/awf.2024.70 (PMC11704570; doi:10.1017/awf.2024.70)
Supplement: Fletcher et al. supplementary material 2 — Fletcher et al. supplementary material [file S0962728624000708sup002.pdf]

Table S1. Further details and signs observed of the 14 animals classified as 'ineffectively stunned' (showing rhythmic breathing and/or at least two of: eyeball rotation, nystagmus, palpebral reflex, corneal reflex) with 0 = absent and 1 = present.

ID	Shot again?	Stun to stick time	Shot in pair	Age category	Stun box behaviour score	Rhythmic breathing	Eyeball rotation	Nystagm us	Corneal reflex	Palpebral reflex	Total signs for ineffective stunning	leg kicking	righting reflex	Failure to collapse	Blinking	Gasping	Vocalisation	Response to cut/knife	TOTAL
1	0	92	0	2.5 - 6yrs	11	1	0	0	0	0	1	1	0	0	0	0	0	NA	2
2	0	80	0	6-12yrs	10	1	0	1	0	0	2	1	0	0	0	0	0	0	3
3	0	56	0	<2.5yrs	10	1	0	0	0	0	1	1	0	0	0	0	0	NA	2
4	0	83	0	12-20yrs	5	1	0	0	0	0	1	1	0	0	0	0	0	NA	2
5	0	72	0	<2.5yrs	7	1	0	1	0	0	2	1	0	0	0	0	0	0	3
6	1	105	0	6-12yrs	11	1	0	1	0	0	2	1	0	0	0	0	0	1	4
7	0	68	0	<2.5yrs	11	1	0	0	0	0	1	1	0	0	0	0	0	0	2
8	0	70	0	<2.5yrs	9	1	0	1	0	0	2	1	0	0	0	0	0	0	3
9	1	NA	1	<2.5yrs	10	1	1	1	1	1	5	1	1	1	0	0	0	NA	8
10	1	NA	1	<2.5yrs	11	1	1	0	0	0	2	0	1	1	0	0	1	NA	5
11	1	92	0	2.5 - 6yrs	12	1	0	0	0	0	1	1	0	0	0	0	0	0	2
12	1	84	1	<2.5yrs	14	1	1	1	1	1	5	1	0	0	1	0	0	0	7
13	0	59	0	<2.5yrs	8	1	0	0	0	0	1	1	0	0	0	0	0	NA	2
14	0	90	0	<2.5yrs	7	1	1	0	0	0	2	1	1	0	0	1	0	0	5
TOTAL	5					14	4	6	2	2	28	13	3	2	1	1	1	1	
